# The nucleolar protein nucleophosmin is essential for autophagy induced by inhibiting Pol I transcription

**DOI:** 10.1038/srep08903

**Published:** 2015-03-10

**Authors:** Naohiro Katagiri, Takao Kuroda, Hiroyuki Kishimoto, Yuki Hayashi, Takuya Kumazawa, Keiji Kimura

**Affiliations:** 1 Graduate School of Life and Environmental Sciences, University of Tsukuba, 1-1-1 Tennnoudai, Tsukuba 305-8577, Japan; 2 Center of Tsukuba Advanced Research Alliance, University of Tsukuba, 1-1-1 Tennnoudai, Tsukuba 305-8577, Japan

## Abstract

Various cellular stresses activate autophagy, which is involved in lysosomal degradation of cytoplasmic materials for maintaining nutrient homeostasis and eliminating harmful components. Here, we show that RNA polymerase I (Pol I) transcription inhibition induces nucleolar disruption and autophagy. Treatment with autophagy inhibitors or siRNA specific for autophagy-related (ATG) proteins inhibited autophagy but not nucleolar disruption induced by Pol I transcription inhibition, which suggested that nucleolar disruption was upstream of autophagy. Furthermore, treatment with siRNA specific for nucleolar protein nucleophosmin (NPM) inhibited this type of autophagy. This showed that NPM was involved in autophagy when the nucleolus was disrupted by Pol I inhibition. In contrast, NPM was not required for canonical autophagy induced by nutrient starvation, as it was not accompanied by nucleolar disruption. Thus, our results revealed that, in addition to canonical autophagy, there may be NPM-dependent autophagy associated with nucleolar disruption.

Eukaryotic cells are continuously exposed to various types of stress; thus, activating an adaptive response to alleviate stress is necessary to maintain cellular homeostasis^1^. One of the key response pathways that removes stress is macroautophagy (hereafter referred to as autophagy)^1^,^2^,^3^,^4^. Autophagy is an intracellular system that degrades cytoplasmic material, such as proteins and organelles, by encircling it in double-membrane vesicles, designated autophagosomes, for delivery to lysosomes^1^,^2^,^3^,^4^. Lysosomes contain a variety of proteases and other acid hydrolases and ultimately degrade this material^1^,^2^,^3^,^4^. In addition, recent reports indicate that selective forms of autophagy, such as mitophagy, pexophagy and nucleophagy, mediate selective removal of mitochondria, peroxisomes and parts of the nucleus, respectively^1^,^5^,^6^,^7^. Autophagy is widely conserved among eukaryotes ranging from yeasts to humans and is strictly regulated by autophagy-related (ATG) proteins^2^,^4^.

Autophagy is induced by various types of stress^1^,^5^. Autophagy is primarily induced by nutrient stress due to depletion of various nutrients, such as amino acids, glucose and growth factors^1^,^3^,^5^. Nutrient stress-induced autophagy degrades cytoplasmic materials and recycles them to maintain nutrient and energy homeostasis, which allows cells to survive under nutrient starvation conditions. For example, yeasts with a deficient autophagy mechanism exhibit poor survival under starvation conditions^8^. Furthermore, mice with knockout of ATG3, ATG5 or ATG7, which are essential for autophagy, die within 1 day after birth, indicating that autophagy is important for mouse survival during the early neonatal starvation period^3^. The studies described below reveal that autophagy is also induced by other types of stress, such as hypoxia, UV irradiation, chemical compounds and heat shock^1^,^3^,^5^. Under these conditions, cells adapt to the stress by activating autophagy to eliminate damaged proteins and organelles^1^,^3^,^5^.

A recent study revealed that the nucleolus, the nuclear component considered to be the site of RNA polymerase I (Pol I)-dependent ribosomal RNA (rRNA) synthesis and a ‘ribosome factory,' acts as a stress sensor^9^,^10^,^11^,^12^,^13^. A number of external and internal insults induce nucleolar stress by disrupting nucleolar structure, which leads to translocation of several nucleolar proteins from the nucleolus to the nucleoplasm, such as nucleophosmin (NPM; also called B23) and nucleostemin and ribosomal proteins, such as RPS7, RPL5, RPL11 and RPL23^11^,^14^,^15^. These translocated proteins cause accumulation and activation of tumour suppressor p53 by interacting with the p53 inhibitor HDM2 and inhibiting HDM2 activity directed towards p53^11^,^14^,^15^. We recently found that a nucleolar protein, Myb-binding protein 1a (MYBBP1A), is anchored to the nucleolus via nucleolar RNA^16^. A number of insults inhibited Pol I transcription and reduced nucleolar RNA levels, which caused MYBBP1A to translocate from the nucleolus to the nucleoplasm^16^. The translocated MYBBP1A activated p53 by enhancing the interaction between p53 and p300, which induced p53 acetylation^16^. Taken together, the nucleolus is regarded as a stress sensor that regulates the location of nucleolar proteins and activates p53 under various stress conditions.

Thus, the nucleolus acts as a stress sensor^9^,^10^,^11^,^12^,^13^, and autophagy is a response to various types of stress^1^,^2^,^3^,^4^. A number of stresses, such as hypoxia, UV irradiation, chemical compounds and heat shock, induce nucleolar disruption^10^,^12^ and autophagy^17^,^18^,^19^,^20^,^21^. Furthermore, nucleolar disruption and autophagy are enhanced in mouse medium spiny neurons by conditional knockout of the RNA Pol I-specific transcription initiation factor-IA (TIF-IA)^22^. A decrease in rRNA synthesis and nucleolar disruption have been reportedly observed in animal models for a variety of neurodegenerative diseases, including Huntington's disease and Parkinson's disease^22^,^23^,^24^,^25^, against which autophagy has protective roles^3^,^26^,^27^. In contrast, increased rRNA synthesis and an enlarged nucleolus are observed in tumour cells^28^,^29^,^30^ with high levels of autophagy^31^,^32^,^33^. Thus, it is speculated that altered nucleolar structure may be related to inducing autophagy.

Here we show that inhibiting Pol I transcription in cells using specific inhibitors and by siRNA treatment induces nucleolar disruption and autophagy. Furthermore, we found that the nucleolar protein NPM played a key role activating autophagy induced by nucleolar disruption. In contrast, NPM was not essential for canonical autophagy induced by nutrient starvation, which was not accompanied by nucleolar disruption.

## Results

### Inhibiting Pol I transcription induces nucleolar disruption and autophagy

**Figure 1:**
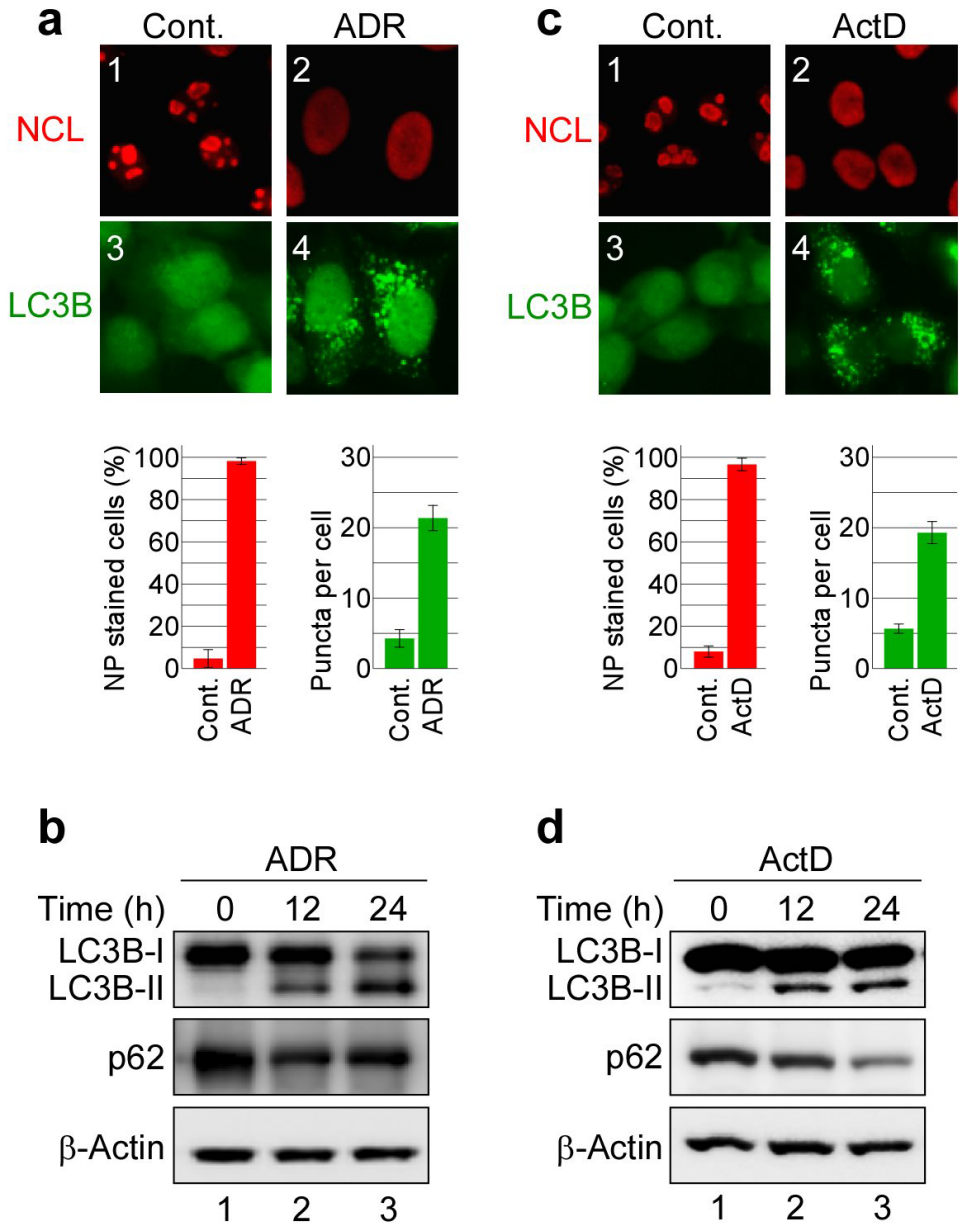
RNA Pol I transcription inhibitors induced nucleolar disruption and autophagy. (a, c) Pol I transcription inhibitors induced nucleolar disruption and the formation of EGFP-LC3B punctate structures. MCF-7/EGFP-LC3B cells were treated with 0.2 μM adriamycin (ADR) (a) or 5 nM actinomycin-D (ActD) (c) for 24 h. The cells were fixed and examined by fluorescence microscopy. The cells were stained with anti-GFP (for LC3B; green) and anti-nucleolin (NCL; red) antibodies to detect LC3B and NCL. The lower left graph shows the percentages of cells with NCL staining in the nucleoplasm (NP). The lower right graph shows the number of EGFP-LC3B puncta per cell. Results are expressed as mean ± standard deviation of triplicate experiments. (b, d) The Pol I transcription inhibitors induced conversion of LC3B-I to LC3B-II and reduced the p62 protein level. MCF-7/EGFP-LC3B cells were treated with 0.2 μM ADR (b) or 5 nM ActD (d) for the indicated times. Cell lysates were prepared and analysed by immunoblotting using anti-GFP (for LC3B-I and LC3B-II), anti-p62 and anti-β-actin antibodies. Complete scans of the blots are presented in Supplementary Figures S11 and S12.

To explore the relationship between nucleolar structure and autophagy, we treated cells with a Pol I transcription inhibitor to induce nucleolar disruption and assessed whether this treatment induced autophagy. To assay for autophagy, we generated MCF-7 cells that stably expressed the enhanced green fluorescent protein (EGFP)-tagged human microtubule-associated protein 1 light chain 3B (LC3B) protein (MCF-7/EGFP-LC3B cells). We treated these cells with 200 nM adriamycin (ADR), which inhibits Pol I transcription and induces autophagy (Fig. 1)^19^,^34^. After ADR treatment, the nucleolar marker protein nucleolin (NCL) was translocated from the nucleolus to the nucleoplasm, which indicated nucleolar disruption (Fig. 1a). Immunostaining of these cells with an anti-GFP antibody showed that ADR treatment enhanced formation of EGFP-LC3B punctate structures, an indicator of autophagy (Fig. 1a and Supplementary Fig. S1a). To further ascertain whether ADR treatment induced autophagy, we assayed for the conversion of LC3B-I to LC3B-II and p62 protein levels by immunoblotting. ADR treatment induced the conversion of LC3B-I to LC3B-II (Fig. 1b) and reduced p62 protein levels (Fig. 1b), both of which are indicators of autophagy. These results show that ADR treatment induced both nucleolar disruption and autophagy.

Furthermore, we tested whether another Pol I transcription inhibitor, actinomycin-D (ActD), could induce nucleolar disruption and autophagy. We treated MCF-7/EGFP-LC3B cells with low-dose ActD (5 nM), which specifically inhibits RNA polymerase I- but not RNA polymerase II- or RNA polymerase III-driven transcription^35^,^36^. As with ADR treatment, ActD induced nucleolar disruption and enhanced formation of EGFP-LC3B punctate structures (Fig. 1c and Supplementary Fig. S1b). In addition, it facilitated the conversion of LC3B-I to LC3B-II and reduced p62 protein levels (Fig. 1d). Taken together, these results indicate that the Pol I transcription inhibitors ADR and ActD induced both nucleolar disruption and autophagy.

**Figure 2:**
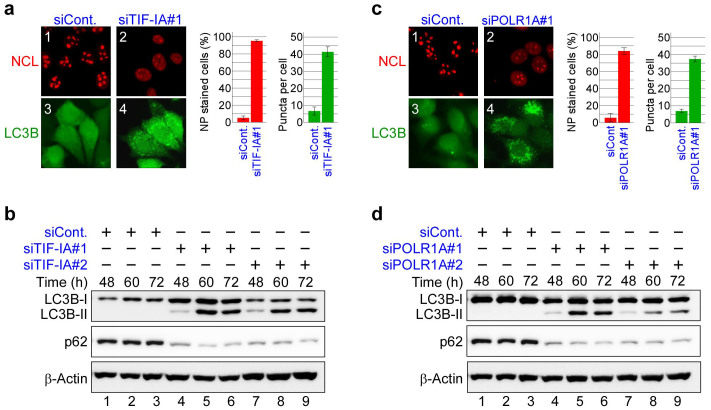
Knockdown of RNA Pol I transcription factors induced nucleolar disruption and autophagy. (a, c) TIF-IA or POLR1A knockdown induced nucleolar disruption and the formation of EGFP-LC3B punctate structures. MCF-7/EGFP-LC3B cells were treated with siRNAs specific for luciferase (siCont), TIF-IA (siTIF-IA#1) or POLR1A (siPOLR1A#1) for 60 h. Immunofluorescent staining and the statistical analysis were performed in the same manner as shown in Figure 1 (a, c). Results are expressed as mean ± standard deviation of triplicate experiments. (b, d) TIF-IA or POLR1A knockdown induced conversion of LC3B-I to LC3B-II and reduced the p62 protein level. MCF-7/EGFP-LC3B cells were treated with siRNAs specific for luciferase (siCont), TIF-IA (siTIF-IA#1 and #2) or POLR1A (siPOLR1A#1 and #2) for the indicated times. Cell lysates were analysed by immunoblotting in the same manner as shown in Figure 1 (b, d). Complete scans of the blots are presented in Supplementary Figures S13 and S14.

ADR and ActD are DNA intercalators that induce DNA breaks by interacting between stacked base pairs^19^,^34^,^35^. Thus, these drugs induce nucleolar disruption and autophagy by inhibiting Pol I transcription or damaging DNA. To distinguish between these possibilities, Pol I transcription was specifically inhibited by treatment with siRNA specific for TIF-IA, a basal Pol I transcription factor^13^,^37^ or siRNA specific for the polymerase (RNA) I polypeptide A (POLR1A), a Pol I catalytic subunit^38^. This finding shows that, in agreement with previous reports, depleting TIF-IA caused nucleolar disruption^13^,^16^, as shown by translocation of the nucleolar marker protein NCL from the nucleolus to the nucleoplasm (Fig. 2a). This treatment inhibited rRNA transcription (Supplementary Fig. S2a) and enhanced p53 protein level (Supplementary Fig. S2b), reflecting the induction of nucleolar stress. The TIF-IA siRNA treatment induced formation of EGFP-LC3B punctate structures (Fig. 2a and Supplementary Fig. S2c). Furthermore, TIF-IA knockdown induced the conversion of LC3B-I to LC3B-II and reduced the p62 protein levels (Fig. 2b). Similar results were found when MCF-7/EGFP-LC3B cells were treated with siRNA specific for POLR1A (Fig. 2 and Supplementary Fig. S2). Taken together, inhibiting Pol I transcription induced both nucleolar disruption and autophagy, suggesting an association between nucleolar disruption and autophagy. However, it is unknown whether nucleolar disruption induces autophagy or vice versa.

### Depleting ATG proteins and autophagy inhibitors repress autophagy, but not nucleolar disruption induced by TIF-IA knockdown

**Figure 3:**
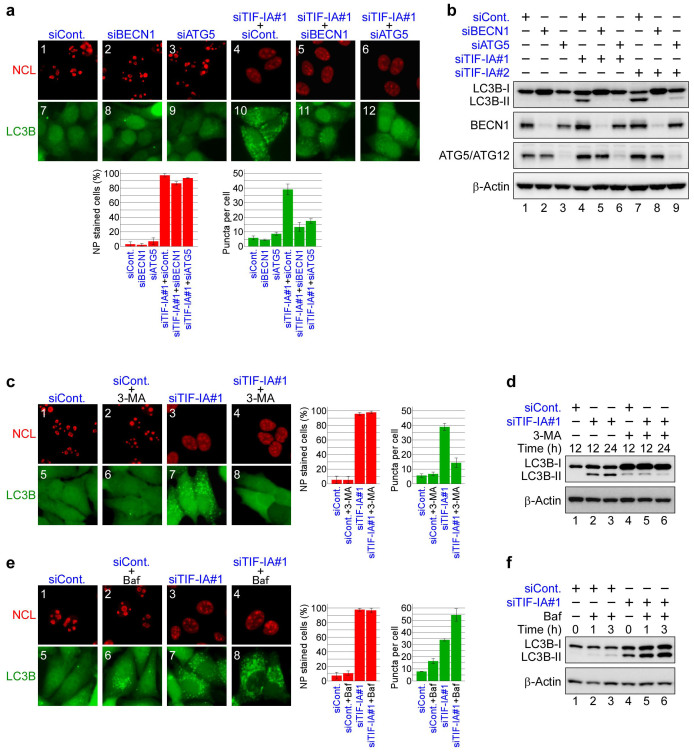
TIF-IA knockdown-dependent autophagy is repressed by autophagy factor knockdown or by autophagy inhibitors. (a) Knocking down BECN1 and autophagy protein 5 (ATG5) repressed formation of the EGFP-LC3B punctate structures dependent on TIF-IA knockdown. MCF-7/EGFP-LC3B cells were treated with combinations of the indicated siRNAs for 60 h. Immunofluorescent staining and the statistical analysis were performed in the same manner as shown in Figure 1. (b) Knocking down BECN1 and ATG5 repressed conversion of LC3B-I to LC3B-II. MCF-7/EGFP-LC3B cells were treated with the indicated siRNAs for 60 h. Cell lysates were prepared and analysed by immunoblot. (c) 3-Methyladenine (3-MA) repressed formation of the EGFP-LC3B punctate structures dependent on TIF-IA knockdown. MCF-7/EGFP-LC3B cells were treated with siCont or siTIF-IA#1 for 36 h before treatment without or with 3 mM 3-MA for 12 h. Immunofluorescent staining and the statistical analysis were performed in the same manner as shown in Figure 1. (d) 3-MA repressed the LC3B-I to LC3B-II conversion dependent on TIF-IA knockdown. MCF-7/EGFP-LC3B cells were treated with the indicated siRNAs for 36 h before treatment without or with 3 mM 3-MA for the indicated times. Cell lysates were prepared and analysed by immunoblot. (e) Bafilomycin A1 enhanced formation of the EGFP-LC3B punctate structures dependent on TIF-IA knockdown. MCF-7/EGFP-LC3B cells were treated with siCont or siTIF-IA#1 for 48 h before treatment without or with 100 nM bafilomycin A1 for 2 h. Immunofluorescent staining and the statistical analysis were performed in the same manner as shown in Figure 1. (f) Bafilomycin A1 enhanced accumulation of the LC3B-II protein dependent on TIF-IA knockdown. MCF-7/EGFP-LC3B cells were treated with the indicated siRNAs for 48 h before treatment without or with 100 nM bafilomycin A1 for the indicated times. Cell lysates were prepared and analysed by immunoblot. Results are expressed mean ± standard deviation of triplicate experiments. Complete scans of the blots are presented in Supplementary Figures S15–S17.

To dissect the autophagy induced by inhibiting Pol I and to investigate the relationship between nucleolar disruption and this type of autophagy in more detail, we depleted the ATG proteins or treated MCF-7/EGFP-LC3B cells with autophagy inhibitors. First, we depleted Beclin 1 (BECN1) and ATG5, both of which are ATG proteins involved in autophagy. BECN1, together with VPS15, VPS34 and Ambra 1, comprise the BECN1 complex, which is essential for allosteric activation of the class III phosphoinositide 3-kinases (PI3-kinase) to generate phosphatidylinositol-3-phoshate required for the initial stages of autophagosome formation^1^,^2^,^39^. ATG5 forms a protein complex with ATG12 and ATG16L1 and catalyses the conversion of LC3-I to LC3-II^1^,^2^,^39^. Knocking down BECN1 or ATG5 repressed the formation of EGFP-LC3B punctate structures induced by TIF-IA siRNA treatment without inhibiting NCL translocation from the nucleolus to the nucleoplasm (Fig. 3a and Supplementary Fig. S3a). BECN1 or ATG5 knockdown also inhibited the conversion of LC3B-I to LC3B-II caused by depleting TIF-IA (Fig. 3b). These results indicate that the ATG proteins BECN1 and ATG5 are involved in autophagy induced by TIF-IA knockdown, as well as other cases of reported autophagy, but that depleting BECN1 or ATG5 was independent of nucleolar disruption.

Furthermore, we tested the effects of autophagy inhibitors on nucleolar disruption and autophagy induced by TIF-IA knockdown. 3-Methyladenine (3-MA), a well-known autophagy inhibitor, inhibits PI3-kinase activity and blocks the initial stages of autophagosome formation^39^. To test whether 3-MA inhibits autophagy induced by TIF-IA knockdown, we treated MCF-7/EGFP-LC3B cells with 3-MA. The formation of EGFP-LC3B punctate structures induced by TIF-IA knockdown was inhibited by 3-MA treatment without inhibiting NCL translocation (Fig. 3c and Supplementary Fig. S3b). Moreover, 3-MA suppressed the conversion of LC3B-I to LC3B-II (Fig. 3d).

Furthermore, we tested the effects of the autophagy inhibitor bafilomycin A1, a specific vacuolar type H^+^-ATPase inhibitor. Bafilomycin A1 prevents the degradation of autophagosomes by inhibiting the fusion of autophagosomes with lysosomes, which results in an apparent accumulation of the autophagy apparatus^4^,^39^. Among TIF-IA siRNA-treated cells, bafilomycin A1 treatment increased the number of cells with the EGFP-LC3B punctate structures but did not affect localisation of NCL in MCF-7/EGFP-LC3B cells (Fig. 3e and Supplementary Fig. S3c). The immunoblotting results showed that bafilomycin A1 increased LC3B-II protein levels in TIF-IA siRNA-treated cells (Fig. 3f). Taken together, treatment with ATG protein siRNAs or autophagy inhibitors, all we tested, influenced autophagy, but did not markedly affect affected nucleolar disruption. Based on these results, nuclear disruption is upstream of autophagy in those cells in which Pol I transcription was inhibited. These results also suggest that inhibiting Pol I induces autophagy in a mechanism similar to that of general autophagy.

### TIF-IA knockdown induces autophagy in the absence of active p53

Several groups, including ours, have reported that nucleolar disruption enhances p53 activity and the expression of multiple p53 target genes^11^,^16^. Furthermore, p53 activation is reportedly involved in activating autophagy in mouse medium spiny neurons^22^. Thus, autophagy was possibly induced by activating p53 when Pol I transcription was inhibited, and the nucleolus was disrupted (Figs. 1 and 2).

**Figure 4:**
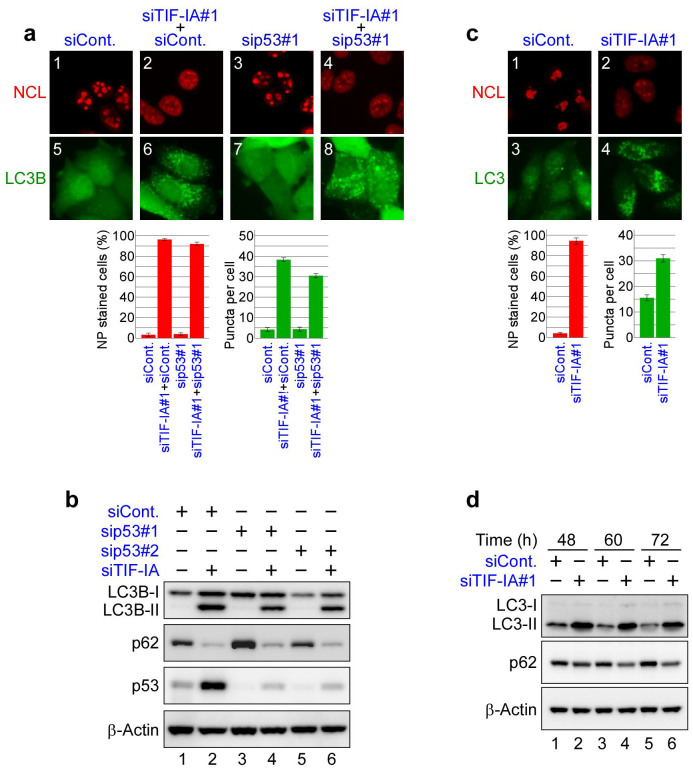
TIF-IA knockdown induces autophagy in the absence of active p53. (a) TIF-IA knockdown induced nucleolar disruption and formation of the LC3B punctate structures in the absence of p53. MCF-7/EGFP-LC3B cells were treated with combinations of indicated siRNAs for 60 h. Immunofluorescent staining and the statistical analysis were performed as shown in Figure 1. (b) TIF-IA knockdown induced conversion of LC3B-I to LC3B-II and reduced the p62 protein level in the absence of p53. MCF-7/EGFP-LC3B cells were treated with siRNAs specific for luciferase (siCont), p53 (sip53#1 and #2) or TIF-IA (siTIF-IA#1) for the indicated times. Cell lysates were analysed by immunoblot. (c) TIF-IA knockdown induced nucleolar disruption and formation of the LC3 punctate structures in HeLa cells. HeLa cells were treated with siCont (panels 1 and 3) or siTIF-IA#1 (panels 2 and 4) for 60 h. The cells were fixed and stained with anti-LC3, which recognises endogenous LC3 proteins (green; panels 1 and 2) and anti-nucleolin (red; panels 3 and 4) antibodies. The statistical analysis was performed in the same manner as shown in Figure 1. Results are expressed as mean ± standard deviation of triplicate experiments. (d) TIF-IA knockdown induced conversion of LC3-I to LC3-II and reduced the p62 protein level in HeLa cells. HeLa cells were treated with siCont or siTIF-IA#1 (siTIF-IA) for 48, 60 and 72 h. Cell lysates were analysed by immunoblot using the indicated antibodies. Complete scans of the blots are presented in Supplementary Figures S18 and S19.

To test whether p53 is essential for autophagy induced by inhibiting Pol I, we treated MCF-7/EGFP-LC3B cells with a combination of p53 siRNA and TIF-IA siRNA (Fig. 4a and b). p53 and TIFI-A double knockdown caused nucleolar disruption, formation of the EGFP-LC3B punctuate structures (Fig. 4a and Supplementary Fig. S4), and the LC3B-I to LC3B-II conversion, as well as reduced p62 levels (Fig. 4b). These results indicate that p53 was dispensable for autophagy induced by inhibiting Pol I in MCF-7/EGFP-LC3B cells.

We then treated HeLa cells, in which p53 is inactivated^40^,^41^, with TIF-IA siRNA and inhibited Pol I transcription (Supplementary Fig. S5a). As a result, nucleolar structures disappeared in these cells after TIF-IA siRNA treatment, as shown by translocation of NCL from the nucleolus to the nucleoplasm (Fig. 4c). Immunostaining of these cells with an anti-LC3 antibody showed that the TIF-IA siRNA treatment induced formation of endogenous LC3 punctate structures (Fig. 4c and Supplementary Fig. S5b). Immunoblotting results showed that the TIF-IA siRNA treatment increased LC3-II levels and reduced p62 protein levels (Fig. 4d and Supplementary Fig. S5c). Taken together with the results of MCF-7/EGFP-LC3B cells depleted of p53 and HeLa cells, autophagy was induced independently of activating p53 when TIF-IA was depleted.

### NPM is required for autophagy induced by inhibiting Pol I

Because inhibiting Pol I transcription induced nucleolar disruption and autophagy, there was a possible relationship between nucleolar disruption and autophagy. One possibility is that some nucleolar proteins that were translocated from the nucleolus induced autophagy. To test this possibility, we screened for nucleolar proteins that were involved in activating autophagy in HeLa cells. We generated a siRNA library directed against mRNAs for nucleolar proteins^42^ and examined the effects of each siRNA on the conversion of LC3-I to LC3-II in cells, treated with TIF-IA siRNA. Based on these screening results, we focused on NPM, which is abundant in various cell types, is a multifunctional protein and is primarily localised to the nucleolus^43^,^44^,^45^,^46^. In addition, a role has not been previously shown for NPM during the induction of autophagy. Thus, we examined NPM function during the induction of autophagy.

**Figure 5:**
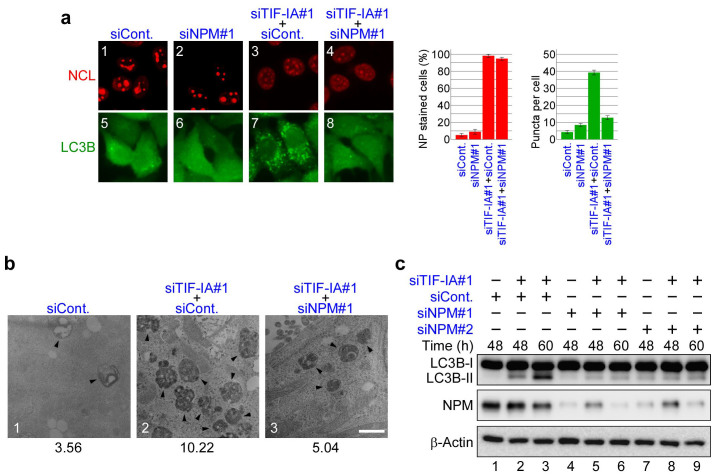
NPM knockdown represses RNA Pol I transcription factor knockdown-dependent autophagy. (a) NPM knockdown repressed formation of the EGFP-LC3B punctate structures dependent on TIF-IA knockdown. MCF-7/EGFP-LC3B cells were treated with siCont (panels 1 and 5), siNPM#1 (panels 2 and 6), siTIF-IA#1 and siCont (panels 3 and 7) or siTIF-IA#1 and siNPM#1 (panels 4 and 8) for 60 h. Immunofluorescent staining and the statistical analysis were performed in the same manner as shown in Figure 1. Results are expressed as mean ± standard deviation of triplicate experiments. (b) Electron micrographs indicate that NPM knockdown repressed autophagy dependent on TIF-IA knockdown. MCF-7 cells were treated with siCont (panel 1), siTIF-IA#1 and siCont (panel 2) or siTIF-IA#1 and siNPM#1 (panel 3) for 60 h, followed by observations under a transmission electron microscope. Autophagic vacuoles (autophagosomes and autolysosomes) are indicated by the arrowheads. Bar, 1 μm. Lower number denotes the percentage of the area of autophagic vacuoles to the cytoplasmic area. (c) NPM knockdown repressed conversion of LC3B-I to LC3B-II dependent on TIF-IA knockdown. MCF-7/EGFP-LC3B cells were treated with siCont (lane 1), siTIF-IA#1 and siCont (lanes 2 and 3), siNPM#1 (lane 4), siTIF-IA#1 and siNPM#1 (lanes 5 and 6), siNPM#2 (lane 7) or siTIF-IA#1 and siNPM#2 (lanes 8 and 9) for the indicated times. Cell lysates were analysed by immunoblot. Complete scans of the blots are presented in Supplementary Figure S20.

We first ascertained that NPM was translocated from the nucleolus when Pol I was inhibited in MCF-7/EGFP-LC3B cells (Supplementary Fig. S6a and Supplementary Fig. S7a), as described in the previous reports^13^,^16^,^22^. To assess whether NPM was involved in the autophagy induced by inhibiting Pol I, we depleted TIF-IA and/or NPM in MCF-7/EGFP-LC3B cells and tested for the formation of EGFP-LC3B punctate structures (Fig. 5a and Supplementary Fig. S6b). The immunostaining results showed that NPM siRNA repressed formation of the EGFP-LC3B punctate structures induced by TIF-IA knockdown (compare panels 7 and 8, Fig. 5a), without affecting NCL translocation (compare panels 3 and 4, Fig. 5a). Furthermore, we conducted a transmission electron microscopy analysis to observe autophagic vacuoles. The electron microscopy analysis showed that TIF-IA knockdown increased the number of autophagic vacuoles, which were suppressed by NPM knockdown (Fig. 5b and Supplementary Fig. S8). Consistent with these immunostaining and electron microscopy results, depleting NPM counteracted the enhanced conversion of LC3B-I to LC3B-II induced by TIF-IA knockdown in MCF-7/EGFP-LC3B cells (Fig. 5c).

Furthermore, we tested for the effect of inhibiting POLR1A (Supplementary Fig. S7). NPM knockdown repressed formation of the EGFP-LC3B punctate structures and the conversion of LC3B-I to LC3B-II induced by POLR1A siRNA treatment (Supplementary Fig. S7b–d). These results were similar to those when TIF-IA siRNA was depleted. Thus, our findings indicate that NPM is essential for the autophagy induced by inhibiting Pol I transcription.

Previous reports showed that NPM enhances p53 accumulation under nucleolar disruption conditions^14^,^28^,^47^. Thus, NPM may have enhanced autophagy by activating p53. However, our results indicate that depleting NPM compromised autophagy induced by depleting TIF-IA without reducing the p53 level (Supplementary Fig. S9). These results suggest that the autophagy induced by NPM was independent of p53 activation.

### NPM knockdown does not affect starvation-induced autophagy

**Figure 6:**
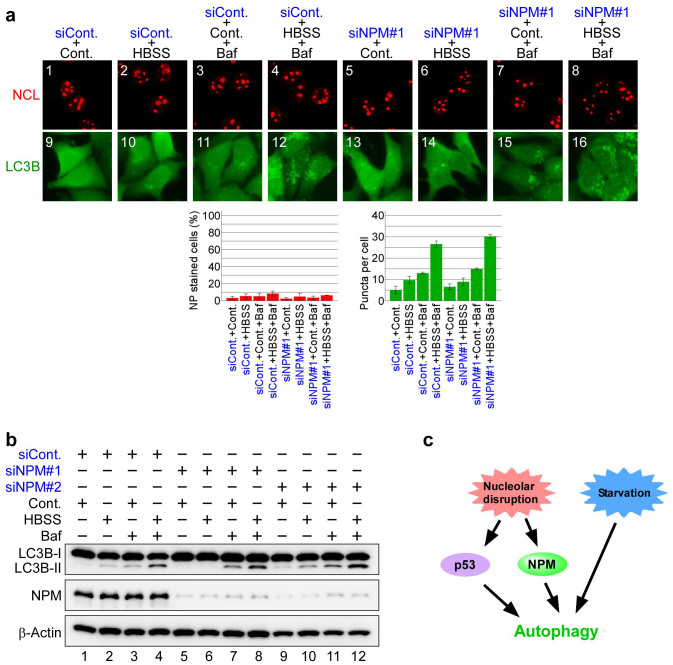
NPM knockdown has little effect on starvation-induced autophagy. (a) NPM knockdown had little effect on formation of the EGFP-LC3B punctate structures induced by starvation. MCF-7/EGFP-LC3B cells were treated with siCont (panels 1–4 and 9–12) or siNPM#1 (panels 5–8) for 60 h. After the siRNA treatment, the cells were cultured in standard medium (Cont.; panels 1, 3, 5, 7, 9, 11, 13 and 15) either without (panels 1, 5, 9 and 13) or with bafilomycin A1 (Baf; panels 3, 7, 11 and 15) or in starvation medium (HBSS; panels 2, 4, 6, 8, 10, 12, 14 and 16) either without (panels 2, 6, 10 and 14) or with bafilomycin A1 (Baf; panels 4, 8, 12 and 16) for 2 h. The cells were fixed and examined by fluorescence microscopy. Immunofluorescent staining and the statistical analysis were performed in the same manner as shown in Figure 1. Results are expressed as mean ± standard deviation of triplicate experiments. (b) NPM knockdown did not affect conversion of LC3B-I to LC3B-II induced by starvation. MCF-7/EGFP-LC3B cells were treated with siCont (lanes 1–4), siNPM#1 (lanes 5–8) or siNPM#2 (lanes 9–12) for 60 h. After siRNA treatment, cells were cultured in standard medium (Cont.; lanes 1, 3, 5, 7, 9 and 11) either without (lanes 1, 5 and 9) or with bafilomycin A1 (Baf; lanes 3, 7 and 11) or in starvation medium (HBSS; panels 2, 4, 6, 8, 10 and 12) either without (lanes 2, 6 and 10) or with bafilomycin A1 (lanes 4, 8 and 12) for 2 h. Cell lysates were analysed by immunoblot. Complete scans of the blots are presented in Supplementary Figure S21. (c) Proposed model for nucleolar disruption-dependent autophagy.

Finally, we tested whether NPM is required for the autophagy induced by cell starvation (Fig. 6 and Supplementary Fig. S10). Nucleolar structures were little affected by serum and amino acid starvation or by treatments with bafilomycin A1 or NPM siRNA (Fig. 6a: panels 1–8). Incubating control siRNA-treated cells in starvation media induced formation of the EGFP-LC3B punctate structures (Fig. 6a: panel 10), which was enhanced in the presence of bafilomycin A1 (Fig. 6a: panel 12). Similar results were obtained with NPM siRNA-treated cells; starvation induced formation of the EGFP-LC3B punctate structures (Fig. 6a: panel 14), which was enhanced by bafilomycin A1 (Fig. 6a: panel 16). To further evaluate the involvement of NPM in starvation-induced autophagy, we examined conversion of LC3B-I to LC3B-II by immunoblotting (Fig. 6b). NPM knockdown had little effect on conversion of LC3B-I to LC3B-II induced by starvation and/or in bafilomycin A1 treated-cells (Fig. 6b). These results indicate that NPM is not involved in the autophagy induced by starvation.

We summarise our findings in Figure 6c. Our experimental results have revealed that nucleolar disruption by inhibiting Pol I transcription induced autophagy. Furthermore, we found that the nucleolar protein NPM is essential for autophagy induced by inhibiting Pol I transcription. In contrast, NPM was not involved in canonical autophagy induced by nutrient starvation. This result suggests that in addition to canonical autophagy, NPM-dependent autophagy induced by Pol I transcription inhibition exists (Fig. 6c).

## Discussion

Recent reports have indicated that the nucleolus is involved in various cellular processes, such as cell cycle regulation, cell proliferation and stress response, in addition to a primary function as the site of ribosome biogenesis^9^,^10^,^11^,^42^. The relationship between the nucleolus and various human diseases has also been suggested^22^,^23^,^28^,^29^. In this study, we found that autophagy was induced when the nucleolus was experimentally disrupted by several methods (Figs. 1 and 2), reflecting the relationship between the nucleolus and autophagy. In addition, we revealed that nucleolar disruption was upstream of autophagy in cells in which Pol I transcription was inhibited using siRNAs for ATG proteins or autophagy inhibitors, because they influenced autophagy without affecting nucleolar disruption (Fig. 3). Recent reports indicate the presence of nucleophagy, which is a selective form of autophagy that removes and degrades damaged or non-essential parts of the nucleus^7^,^48^. We found no evidence of nucleophagy when the nucleolus was disrupted; however, it is intriguing to test for the presence of nucleophagy because Pol I inhibition may influence nuclear structures, including nucleolar dynamics.

There are two possible mechanisms by which nucleolar disruption could induce autophagy; one is that reduced protein synthesis via a reduced number of ribosomes causes autophagy^9^,^13^, and the other is that translocation of nucleolar proteins causes autophagy^11^,^15^. The former mechanism is unlikely because cycloheximide, an inhibitor of protein synthesis, does not enhance autophagy^49^,^50^. To test the latter possibility, we screened for nucleolar proteins, the knockdown of which compromised autophagy induced by Pol I inhibition. On the basis of these results, we focused on NPM, which is abundant in various cell types, and is a multifunctional protein localised primarily to the nucleolus^43^,^44^,^45^,^46^.

NPM knockdown inhibited autophagy without affecting nucleolar disruption when Pol I transcription factors were depleted (Fig. 5 and Supplementary Fig. S7). Furthermore, NPM was not essential for canonical autophagy induced by nutrient starvation stress, which did not induce nucleolar disruption (Fig. 6a and b). On the basis of this result, NPM may have induced autophagy when nucleolar structure was disrupted by Pol I inhibition, a mechanism different from that involved in canonical autophagy (Fig. 6c). A previous study showed that p53 is implicated in autophagy induced by nucleolar stress^22^. Thus, NPM may enhance autophagy by activating p53, as NPM enhances the accumulation and activation of p53^14^,^47^. However, our screening results showed that NPM was also involved in autophagy of HeLa cells, which are cells that do not have active p53. Furthermore, NPM knockdown compromised autophagy without reducing the p53 level (Supplementary Fig. S9). On the basis of these results, it is likely that NPM-induced autophagy is independent of p53 activation under our conditions. Thus, NPM- and p53-dependent pathways may be present independently in the autophagy induced by nucleolar disruption (Fig. 6c).

Autophagy is reportedly related to various diseases, such as neurodegenerative diseases and tumours^3^,^26^,^27^,^31^,^32^,^33^. In addition, abnormalities in nucleolar structure are observed in such diseases^22^,^23^,^24^,^25^,^28^,^29^,^30^. A decrease in rRNA transcription and reduced nucleolar volume are found in animal models of various neurodegenerative diseases^22^,^23^,^24^,^25^. Furthermore, targeted disruption of the nucleolus by conditional TIF-IA ablation results in a mouse model with neurodegenerative disease-like phenotypes^22^,^24^. Thus, nucleolar stress and its resultant shrinking nucleolus is one of the causes of neurodegenerative diseases. Several lines of evidence indicate that autophagy has neuroprotective functions^3^,^26^,^27^. For example, disruption of the nucleolus in response to nucleolar stress activates p53, which enhances autophagy and prolongs neural survival, along with PTEN^22^; although, the effect of p53 on neural survival is biphasic; p53 causes cell death by inducing apoptosis^23^. Moreover, neural cell-specific Atg5 or Atg7 conditional knockout mice develop neurodegenerative diseases^51^,^52^, indicating the importance of autophagy to prevent such diseases. In addition, NPM translocates from the nucleolus to the nucleoplasm in response to nucleolar stress^12^,^13^. Overexpression of NPM is neuroprotective in cellular models^53^. Taken together with our results showing that NPM has a critical role in the induction of autophagy by nucleolar disruption (Fig. 5 and Supplementary Fig. S7), NPM may have a role in alleviating neuronal diseases by inducing autophagy. Thus, NPM could be a molecular drug target or be used in a strategy to cure neuronal disease.

Next, we discuss tumours. An increase in rRNA synthesis and an enlarged nucleolus are tumour hallmarks^28^,^29^,^30^, in contrast to neurodegenerative diseases. Autophagy is enhanced in tumour cells^31^,^32^,^33^; however, the link between autophagy and the nucleolus has not been established. We propose that NPM could be a key molecule. NPM is overexpressed in various tumour types, including gastric, colon, ovarian and prostate^43^,^54^,^55^. NPM promotes tumour progression by enhancing cell proliferation and inhibiting cell differentiation and apoptosis^43^,^54^,^55^. Translocation of NPM to the cytoplasm, which is caused by a mutation in the NPM nucleolar localisation domain, is detected in >30% of primary acute myelogenous leukaemia cells^56^,^57^. Thus, translocation of NPM may enhance cancer progression by facilitating autophagy, which is related to tumorigenesis; autophagy suppresses tumour growth during the initiation stage, although it also plays a critical role in tumour progression and maintenance^58^,^59^,^60^. If this is the case, then inhibiting autophagy would enhance the effect of anti-cancer drugs in tumours with translocated NPM, as inhibiting autophagy enhances the cytotoxic effects of some anti-cancer agents^31^,^32^,^33^,^60^. Alternatively, inhibition or downregulation of NPM could lead to a new cancer therapy strategy.

## Methods

### Cell culture

MCF-7 human breast cancer cells and HeLa human cervical cancer cells were maintained in DMEM (Sigma, St. Louis, MO, USA) as previously described^16^.

### Plasmids

Full-length cDNA encoding human *LC3B* was amplified by polymerase chain reaction (PCR) using total DNA isolated from MCF-7 cells. PCR amplification was conducted using the following primers: human LC3B forward, 5′-GGCGAATTCGATGCCGTCGGAGAAGACCTTC-3′ and reverse, 5′-GGCGTCGACTTACACTGACAATTTCATCCC-3′. cDNA was cloned into the pEGFP-C1 vector (Invitrogen, Carlsbad, CA, USA) to generate the pEGFP-C1-LC3B plasmid.

### Generation of stable cell lines

MCF-7 cells were transfected with the pEGFP-C1-LC3B plasmid using Lipofectamine LTX & Plus Reagent (Invitrogen) according to the manufacturer's guidelines. Selective pressure was applied 48 h after transfection using DMEM containing 0.5 mg/ml G418. At least five stable transgenic clones were isolated after 14 days.

### siRNA transfection

siRNA transfection was performed as previously described^16^. In brief, Cells at 30–50% confluence were transfected with 20 nM siRNA (see below) using Lipofectamine RNAiMAX (Invitrogen) for 48–72 h according to the manufacturer's protocol.

### Antibodies

The anti-GFP, anti-p62/SQSTM1, anti-BECN1, anti-ATG5 and anti-LC3 antibodies were purchased from MBL International (Woburn, MA, USA) (598, PM045, PM017, M153-3 and PM036, respectively). Anti-β-actin and anti-p53 antibodies were purchased from Santa Cruz Biotechnology (Santa Cruz, CA, USA) (sc-47778 and sc-126, respectively). The anti-NCL antibody was purchased from Abcam (Cambridge, MA, USA) (ab-13541) and anti-NPM antibody was purchased from Invitrogen (32-5200).

### Immunofluorescent staining

Cells grown on chamber slides were washed with phosphate-buffered saline (PBS; 140 mM NaCl, 2.7 mM KCl, 1.5 mM KH_2_PO_4_ and 8.1 mM Na_2_HPO_4_) and fixed in 4% paraformaldehyde phosphate buffer solution (Wako Pure Chemical, Osaka, Japan) for 10 min. After washing twice with PBS, the cells were permeabilised in 0.1% Triton X-100 in PBS and blocked with 0.1% Triton X-100 in PBS containing 1% bovine serum albumin and 10% foetal bovine serum for 1 h at room temperature. Then the MCF-7/EGFP-LC3B cells were incubated with anti-GFP, anti-NCL or anti-NPM antibodies, and HeLa cells were incubated with anti-LC3, anti-NCL or anti-NPM antibodies for 6 h at 4°C. After washing with 0.1% Triton X-100 in PBS, the cells were incubated with Alexa Fluor 488- and 594-conjugated secondary antibodies (Invitrogen) for 1 h and mounted with Vectashield (Vector Laboratories, Burlingame, CA, USA). Immunofluorescence signals were detected by Biorevo BZ-9000 immunofluorescence microscopy (Keyence, Osaka, Japan). More than 150 cells were determined in triplicate experiments to quantify the number of EGFP-LC3B and LC3 puncta. More than 100 cells were determined in triplicate experiments to quantify the number of cells with translocated NCL.

### siRNA

All siRNAs were purchased from Invitrogen. The following sequences were used: human TIF-IA#1-siRNA sense: 5′-CGACACCGUGGUUUCUCAUGCCAAU-3′ and antisense: 5′-AUUGGCAUGAGAAACCACGGUGUCG-3′; human TIF-IA#2-siRNA sense: 5′-AGGAUGUCUGCUAUGUAGAUGGUAA-3′ and antisense, 5′-UUACCAUCUACAUAGCAGACAUCCU-3′; human POLR1A#1-siRNA sense: 5′-CCUAGGAGACCAGAUGUUUACUAAU-3′ and antisense: 5′-AUUAGUAAACAUCUGGUCUCCUAGG-3′; human POLR1A#2-siRNA sense: 5′-CAACAGCAAGUUGACUAUCACGUUU-3′ and antisense: 5′-AAACGUGAUAGUCAACUUGCUGUUG-3′; human BECN1-siRNA sense: 5′-CCACUCUGUGAGGAAUGCACAGAUA-3′ and antisense: 5′-UAUCUGUGCAUUCCUCACAGAGUGG-3′; human ATG5-siRNA sense: 5′-CAAAGAAGUUUGUCCUUCUGCUAUU-3′ and antisense: 5′-AAUAGCAGAAGGACAAACUUCUUUG-3′; human p53#1-siRNA sense: 5′- UUCCGUCCCAGUAGAUUACCACUGG-3′ and antisense: 5′- CCAGUGGUAAUCUACUGGGACGGAA-3′; human p53#2-siRNA sense: 5′- GCUUCGAGAUGUUCCGAGAGCUGAA-3′ and antisense: 5′- UUCAGCUCUCGGAACAUCUCGAAGC-3′; human NPM1#1-siRNA sense: 5′-GAUGACUGACCAAGAGGCUAUUCAA-3′ and antisense: 5′-UUGAAUAGCCUCUUGGUCAGUCAUC-3′ and human NPM1#2-siRNA sense: 5′-UGUAUGGAAUGUUAUGAUAGGACAU-3′ and antisense: 5′-AUGUCCUAUCAUAACAUUCCAUACA-3′. The Stealth™ RNAi Luciferase Reporter Control Duplex was used as a control.

### Immunoblotting

Immunoblotting was performed as previously described^16^ with minor modifications. The membranes were blocked with 3% skim milk in TBS-T buffer (20 mM Tris-HCl, pH 7.5; 150 mM NaCl and 0.05% Tween20) for 30 min.

### Electron microscopy

MCF-7 cells were prefixed in 2% glutaraldehyde in 0.1 M phosphate buffer at 4°C. The following experiments were performed by the Hanaichi Ultra Structure Institute. In brief, the cells were washed overnight in 0.1 M phosphate buffer at 4°C and were postfixed in 2% osmium tetraoxide for 3 h. The cells were further dehydrated in a graded ethanol series and embedded in epoxy resin. Ultrathin sections were stained with 2% uranyl acetate and lead staining solution and observed with a JEM-1200EX electron microscope. At least 100 μm^2^ of the cytoplasmic area was determined to quantify the ratio of the area of autophagic vacuoles to the cytoplasmic area.

## Author Contributions

N.K., T. Kuro., H.K., Y.H., T. Kuma. and K.K. designed the experiments. Most experiments were performed by N.K.. N.K. and K.K. wrote the manuscript.

## Supplementary Material

Supplementary Information Supplementary Information
