# Supplementary material for: The nucleolar protein nucleophosmin is essential for autophagy induced by inhibiting Pol I transcription
Source: Sci Rep. 2015 Mar 10;5:8903. doi: 10.1038/srep08903 (PMC4354046; doi:10.1038/srep08903)
Supplement: Supplementary Information [file srep08903-s1.pdf]

Supplementary information

The nucleolar protein nucleophosmin is essential for autophagy induced by inhibiting Pol I transcription

Naohiro Katagiri[1], Takao Kuroda[2], Hiroyuki Kishimoto[1, 2], Yuki Hayashi[1], Takuya Kumazawa[1] & Keiji Kimura[1, *]

[1]Graduate School of Life and Environmental Sciences, University of Tsukuba, 1-1-1 Tennnoudai, Tsukuba 305-8577, Japan

[2]Center of Tsukuba Advanced Research Alliance, University of Tsukuba, 1-1-1 Tennnoudai, Tsukuba 305-8577, Japan

[*]Corresponding author

RNA purification and RT-qPCR

Total RNA was isolated from cultured cells using Sepasol RNA I Super reagent (Nacalai tesque, Kyoto, Japan) and subjected to reverse transcription using PrimeScript RT-PCR kit (TaKaRa, Shiga, Japan) according to the manufacturer's protocol. RT–qPCR was performed using the Thermal Cycler Dice Real Time System (TaKaRa) and Platinum SYBR Green qPCR SuperMix (Invitrogen, Carlsbad, CA, USA). For PCR amplification, the specific primers 5′- CCAACCGCGAGAAGATGA -3′ and 5′- CCAGAGGCGTACAGGGATAG -3′ for human β-actin, 5′-GAACGGTGGTGTGTCGTTC-3′, and 5′-GCGTCTCGTCTCGTCTCACT-3′ for pre-rRNA were used. Results were normalized to β-actin.

Supplementary Figure S1 RNA Pol I transcription inhibitors increase the number of EGFP-LC3B puncta. MCF-7/EGFP-LC3B cells were treated with 0.2 μM adriamycin (ADR) (a) or 5 nM actinomycin-D (ActD) (b) for 24 h. The cells were fixed and stained with anti-GFP for LC3B. The histograms denote the number of EGFP-LC3B puncta. More than 150 cells were determined in triplicate experiments.

Supplementary Figure S2 Knockdown of RNA Pol I transcription factors causes rRNA transcription inhibition, p53 accumulation and autophagy. (a, b, d, e) MCF-7/EGFP-LC3B cells were treated with siRNAs specific for luciferase (siCont), TIF-IA (siTIF-IA#1 and #2) or POLR1A (siPOLR1A#1 and #2) for 60 h. (c, f) MCF-7/EGFP-LC3B cells were treated with siRNAs specific for luciferase (siCont), TIF-IA (siTIF-IA#1) or POLR1A (siPOLR1A#1) for 60 h. (a, d) TIF-IA or POLR1A knockdown reduced rRNA transcription. pre-rRNA levels were quantified by RT-qPCR. Results are expressed as mean ± standard deviation of triplicate experiments. (b, e) TIF-IA or POLR1A knockdown increased p53 protein levels. Cell lysates were analysed by immunoblotting. (c, f) TIF-IA or POLR1A knockdown increased the number of EGFP-LC3B puncta. Cells were fixed and stained with anti-GFP for LC3B. The histograms denote the number of EGFP-LC3B puncta. More than 150 cells were determined in triplicate experiments.

Supplementary Figure S3 TIF-IA knockdown-dependent autophagy is repressed by autophagy factor knockdown or by autophagy inhibitors. (a) Knocking down BECN1 and ATG5 repressed formation of the EGFP-LC3B punctate structures dependent on TIF-IA knockdown. MCF-7/EGFP-LC3B cells were treated with

combinations of the indicated siRNAs for 60 h. The cells were fixed and stained with anti-GFP for LC3B. The histograms denote the number of EGFP-LC3B puncta. More than 150 cells were determined in triplicate experiments. (b) 3-Methyladenine (3-MA) repressed formation of the EGFP-LC3B punctate structures dependent on TIF-IA knockdown. MCF-7/EGFP-LC3B cells were treated with siCont or siTIF-IA#1 for 36 h before treatment without or with 3 mM 3-MA for 12 h. The statistical analysis was performed as shown in (a). (c) Bafilomycin A1 enhanced formation of the EGFP-LC3B punctate structures dependent on TIF-IA knockdown. MCF-7/EGFP-LC3B cells were treated with siCont or siTIF-IA#1 for 48 h before treatment without or with 100 nM bafilomycin A1 for 2 h. The statistical analysis was performed as shown in (a).

Supplementary Figure S4 TIF-IA knockdown increases the number of EGFP-LC3B puncta in the absence of p53. MCF-7/EGFP-LC3B cells were treated with combinations of siRNAs specific for TIF-IA and p53 for 60 h. The cells were fixed and stained with anti-GFP for LC3B. The histograms denote the number of EGFP-LC3B puncta. More than 150 cells were determined in triplicate experiments.

Supplementary Figure S5 TIF-IA knockdown induces autophagy in HeLa cells. (a) TIF-IA knockdown reduced rRNA transcription in HeLa cells. HeLa cells were treated with siRNAs specific for luciferase (siCont) and TIF-IA (siTIF-IA#1 and #2) for 60 h. pre-RNA levels were quantified by RT-qPCR. Results are expressed as mean ± standard deviation of triplicate experiments. (b) TIF-IA knockdown increased formation of the EGFP-LC3B punctate structures. HeLa cells were treated with the indicated siRNAs for 60 h. The cells were fixed and stained with anti-GFP for LC3B. The histograms denote

the number of EGFP-LC3B puncta. More than 150 cells were determined in triplicate experiments. (c) TIF-IA knockdown reduced p62 protein levels in HeLa cells. HeLa cells were treated with siCont or siTIF-IA#1 for 48, 60 and 72 h. Cell lysates were analysed by immunoblot. The graph indicates relative protein levels of siTIF-IA-treated cells to those of siCont cells at each time.

Supplementary Figure S6 NPM knockdown represses autophagy induced by TIF-IA knockdown. (a) NPM was translocated from the nucleolus by TIF-IA knockdown. MCF-7/EGFP-LC3B cells were treated with siRNAs specific for luciferase (siCont) and TIF-IA (siTIF-IA#1 and #2) for 60 h. The cells were fixed and stained with anti-NPM. (b) NPM knockdown repressed formation of the EGFP-LC3B punctate structures dependent on TIF-IA knockdown. MCF-7/EGFP-LC3B cells were treated with the indicated siRNA for 60 h. Cells were fixed and stained with anti-GFP for LC3B. The histograms denote the number of EGFP-LC3B puncta. More than 150 cells were determined in triplicate experiments.

Supplementary Figure S7 NPM knockdown represses autophagy induced by POLR1A knockdown. (a) NPM was translocated from the nucleolus by POLR1A knockdown. MCF-7/EGFP-LC3B cells were treated with siRNAs specific for luciferase (siCont) or POLR1A (siPOLR1A#1 and #2) for 60 h. The cells were fixed and stained with anti-NPM. (b) NPM knockdown repressed formation of the EGFP-LC3B punctate structures dependent on POLR1A knockdown. MCF-7/EGFP-LC3B cells were treated with combinations of the indicated siRNAs for 60 h. A statistical analysis was performed in the same manner as shown in Figure 1. Results are expressed as mean ±

standard deviation of triplicate experiments. (c) NPM knockdown repressed formation of the EGFP-LC3B punctate structures dependent on POLR1A knockdown. MCF-7/EGFP-LC3B cells were treated with the indicated siRNA for 60 h. The cells were fixed and stained with anti-GFP for LC3B. The histograms denote the number of GFP-LC3B puncta. More than 150 cells were determined in triplicate experiments. (d) NPM knockdown repressed conversion of LC3B-I to LC3B-II dependent on POLRIA knockdown. MCF-7/EGFP-LC3B cells were treated with combinations of the indicated siRNAs for the indicated times. The cell lysates were analysed by immunoblot.

Supplementary Figure S8 Electron micrographs indicate that NPM knockdown repressed autophagy dependent on TIF-IA knockdown. Enlarged images of Figure 5b. MCF-7 cells were treated with siCont (panel 1), siTIF-IA#1 and siCont (panel 2) or siTIF-IA#1 and siNPM#1 (panel 3) for 60 h, followed by observations under a transmission electron microscope. Bar, 1 μm.

Supplementary Figure S9 NPM knockdown has little effect on p53 protein level. NPM knockdown did not reduce p53 level. MCF-7/EGFP-LC3B cells were treated with siCont, siNPM#1 or siNPM#2 either without or with TIF-IA#1 for 60 h. The cell lysates were analysed by immunoblot using the indicated antibodies.

Supplementary Figure S10 NPM knockdown has little effect on starvation-induced autophagy. (a) NPM did not translocate from the nucleolus by starvation. MCF-7/EGFP-LC3B cells were cultured in standard medium (Cont.; panels 1, 3, 5 and 7) either without (panels 1 and 5) or with bafilomycin A1 (Baf; panels 3 and 7) or in

starvation medium (HBSS; panels 2, 4, 6 and 8) either without (panels 2 and 6) or with bafilomycin A1 (panels 4 and 8) for 2 h. The cells were fixed and examined by fluorescence microscopy. The cells were fixed and stained with anti-NPM. (b) NPM knockdown had little effect on formation of the EGFP-LC3B punctate structures induced by starvation. MCF-7/EGFP-LC3B cells were treated with siCont (lanes 1–4) or siNPM#1 (panels 5–8) for 60 h. After the siRNA treatment, the cells were cultured in standard medium (Cont.; lanes 1, 3, 5 and 7) either without (lanes 1 and 5) or with bafilomycin A1 (Baf; lanes 3 and 7) or in starvation medium (lanes; 2, 4, 6 and 8) either without (lanes 2 and 6) or with bafilomycin A1 (lanes 4 and 8) for 2 h. The cells were fixed and stained with anti-GFP for LC3B. The histograms denote the number of EGFP-LC3B puncta. More than 150 cells were determined in triplicate experiments.

Supplementary Figure S11 complete scan of the blots presented in Figure 1b.

Supplementary Figure S12 complete scan of the blots presented in Figure 1d.

Supplementary Figure S13 complete scan of the blots presented in Figure 2b.

Supplementary Figure S14 complete scan of the blots presented in Figure 2d.

Supplementary Figure S15 complete scan of the blots presented in Figure 3b.

Supplementary Figure S16 complete scan of the blots presented in Figure 3d.

Supplementary Figure S17 complete scan of the blots presented in Figure 3f.

Supplementary Figure S18 complete scan of the blots presented in Figure 4b.

Supplementary Figure S19 complete scan of the blots presented in Figure 4d.

Supplementary Figure S20 complete scan of the blots presented in Figure 5c.

Supplementary Figure S21 complete scan of the blots presented in Figure 6b.

a

Histogram of Figure 1a

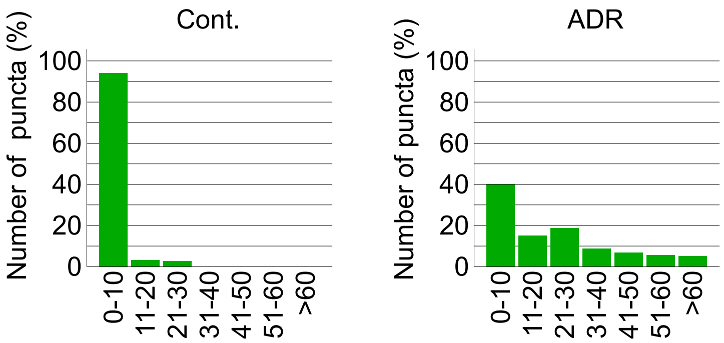

b

Histogram of Figure 1c

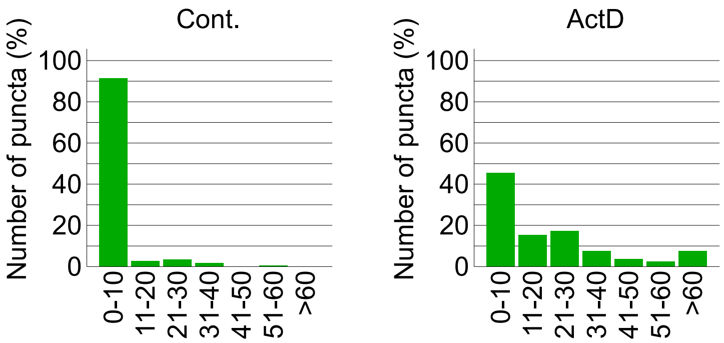

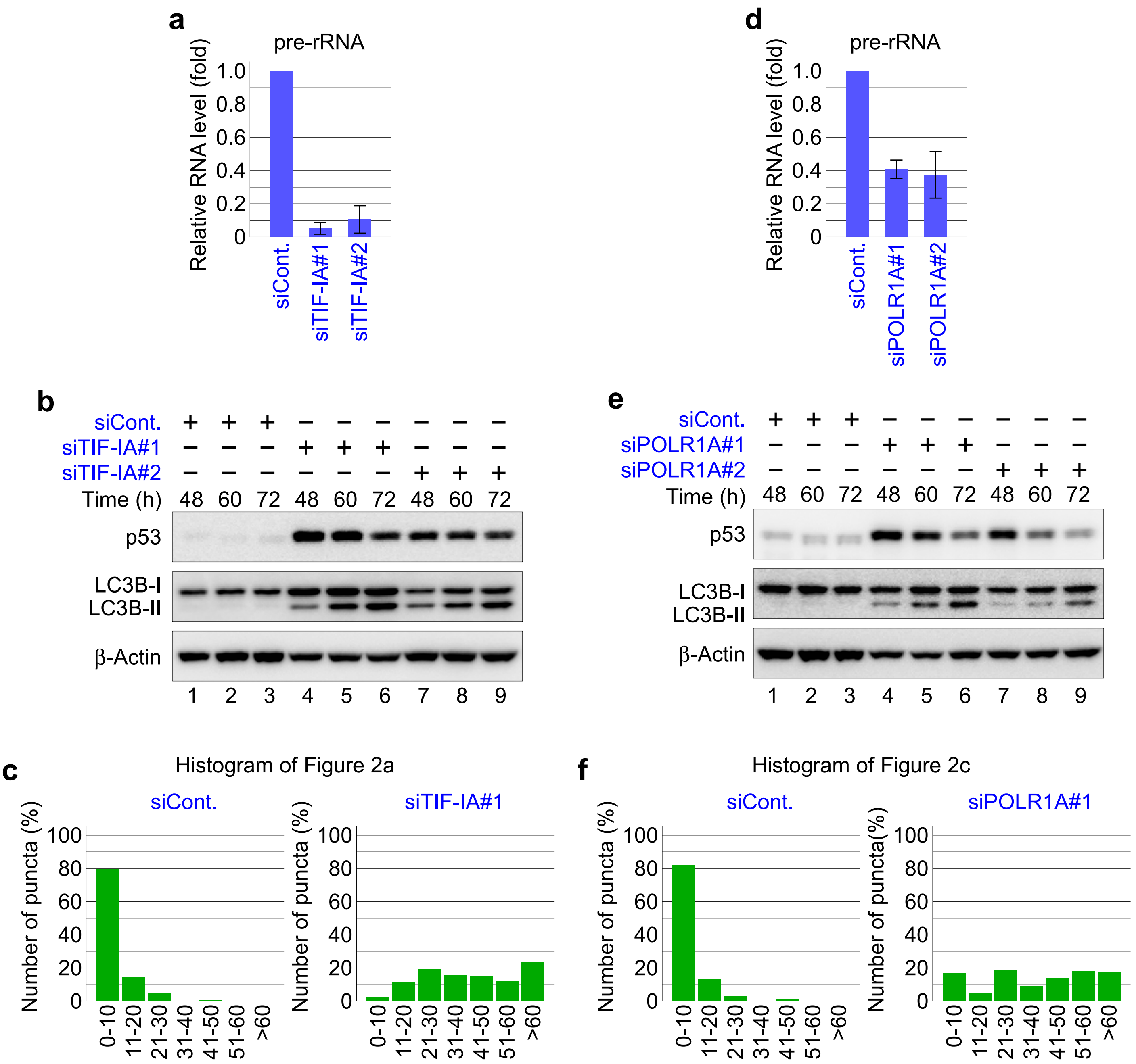
a
pre-rRNA
Relative RNA level (fold)
1.0
0.8
0.6
0.4
0.2
0
siCont.
siTIF-IA#1
siTIF-IA#2
d
pre-rRNA
Relative RNA level (fold)
siCont.
siPOLR1A#1
siPOLR1A#2
b
siCont.
siTIF-IA#1
siTIF-IA#2
Time (h)
48 60 72 48 60 72 48 60 72
p53
LC3B-I
LC3B-II
β-Actin
1 2 3 4 5 6 7 8 9
e
siCont.
siPOLR1A#1
siPOLR1A#2
Time (h)
48 60 72 48 60 72 48 60 72
p53
LC3B-I
LC3B-II
β-Actin
1 2 3 4 5 6 7 8 9
c
Histogram of Figure 2a
siCont.
siTIF-IA#1
Number of puncta (%)
100
80
60
40
20
0
0-10
11-20
21-30
31-40
41-50
51-60
>60
f
Histogram of Figure 2c
siCont.
siPOLR1A#1
Number of puncta (%)
Number of puncta(%)

Histogram of Figure 3a

a

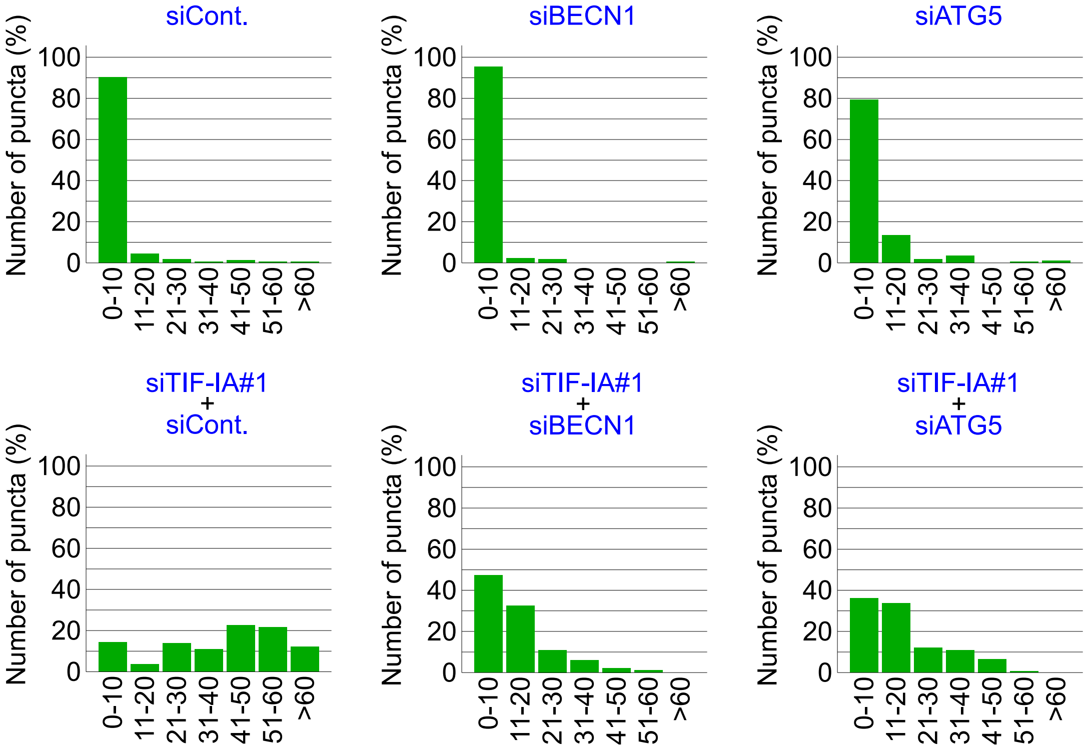

Histogram of Figure 3c

b

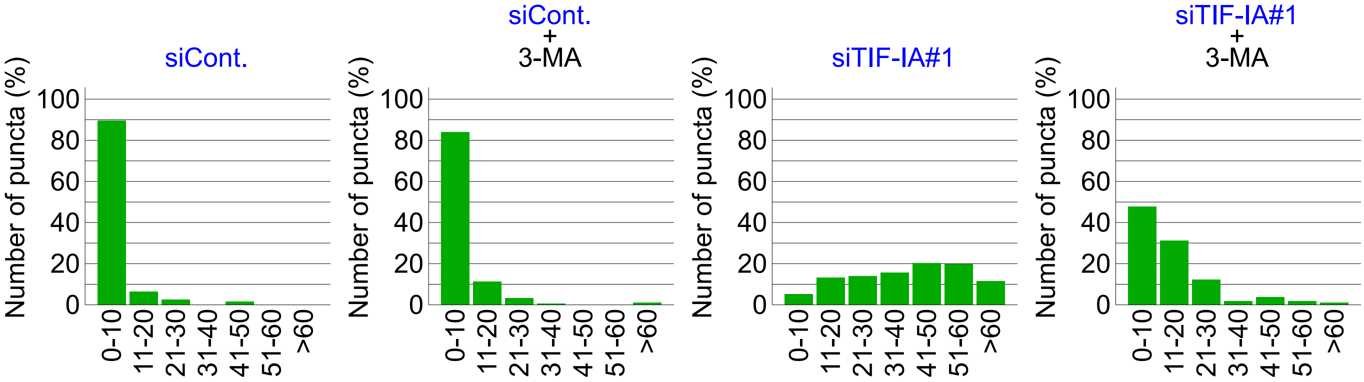

Histogram of Figure 3e

c

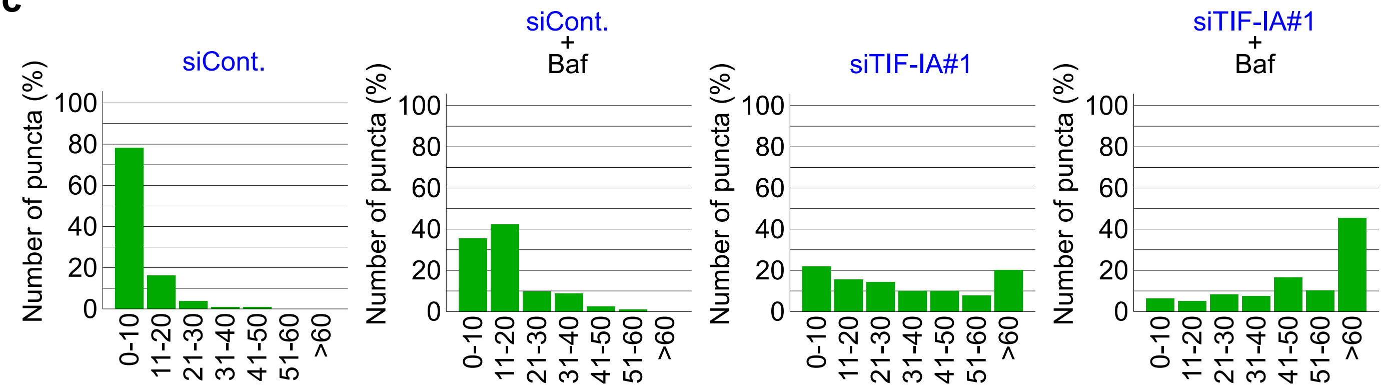

Histogram of Figure 4a

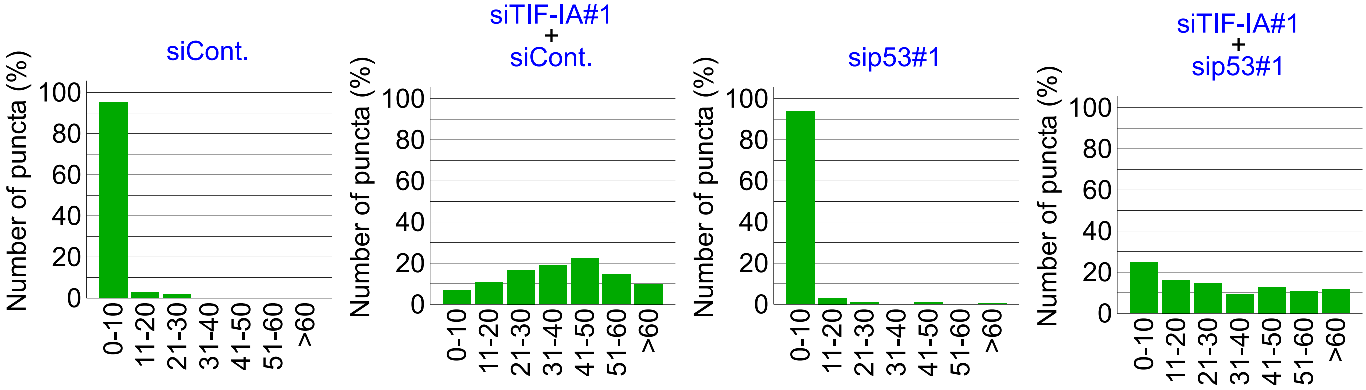

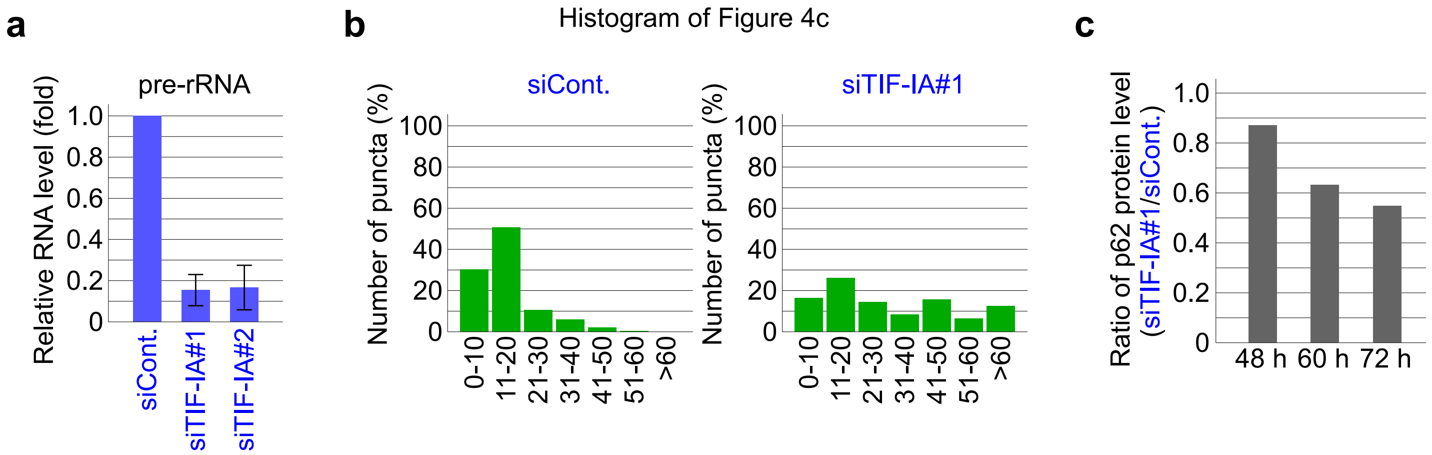
a
pre-rRNA
Relative RNA level (fold)
1.0
0.8
0.6
0.4
0.2
0
siCont.
siTIF-IA#1
siTIF-IA#2
b
Histogram of Figure 4c
siCont.
siTIF-IA#1
Number of puncta (%)
100
80
60
40
20
0
0-10
11-20
21-30
31-40
41-50
51-60
>60
c
Ratio of p62 protein level
(siTIF-IA#1/siCont.)
1.0
0.8
0.6
0.4
0.2
0
48 h
60 h
72 h

a

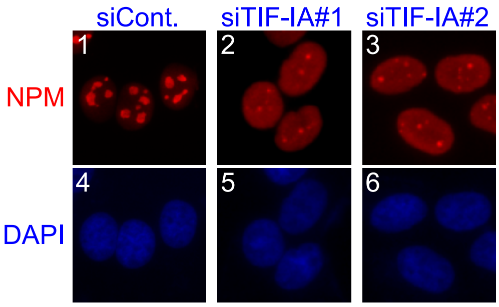

Histogram of Figure 5a

b

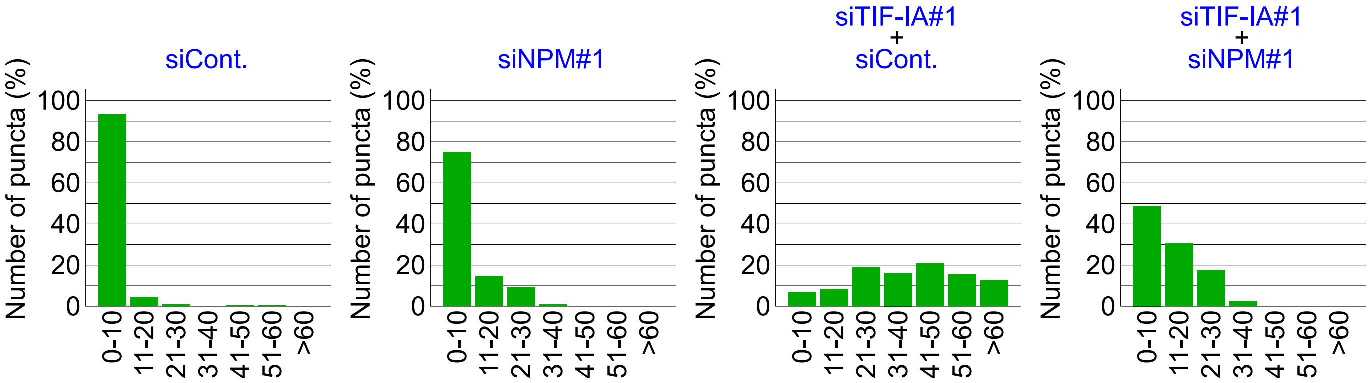

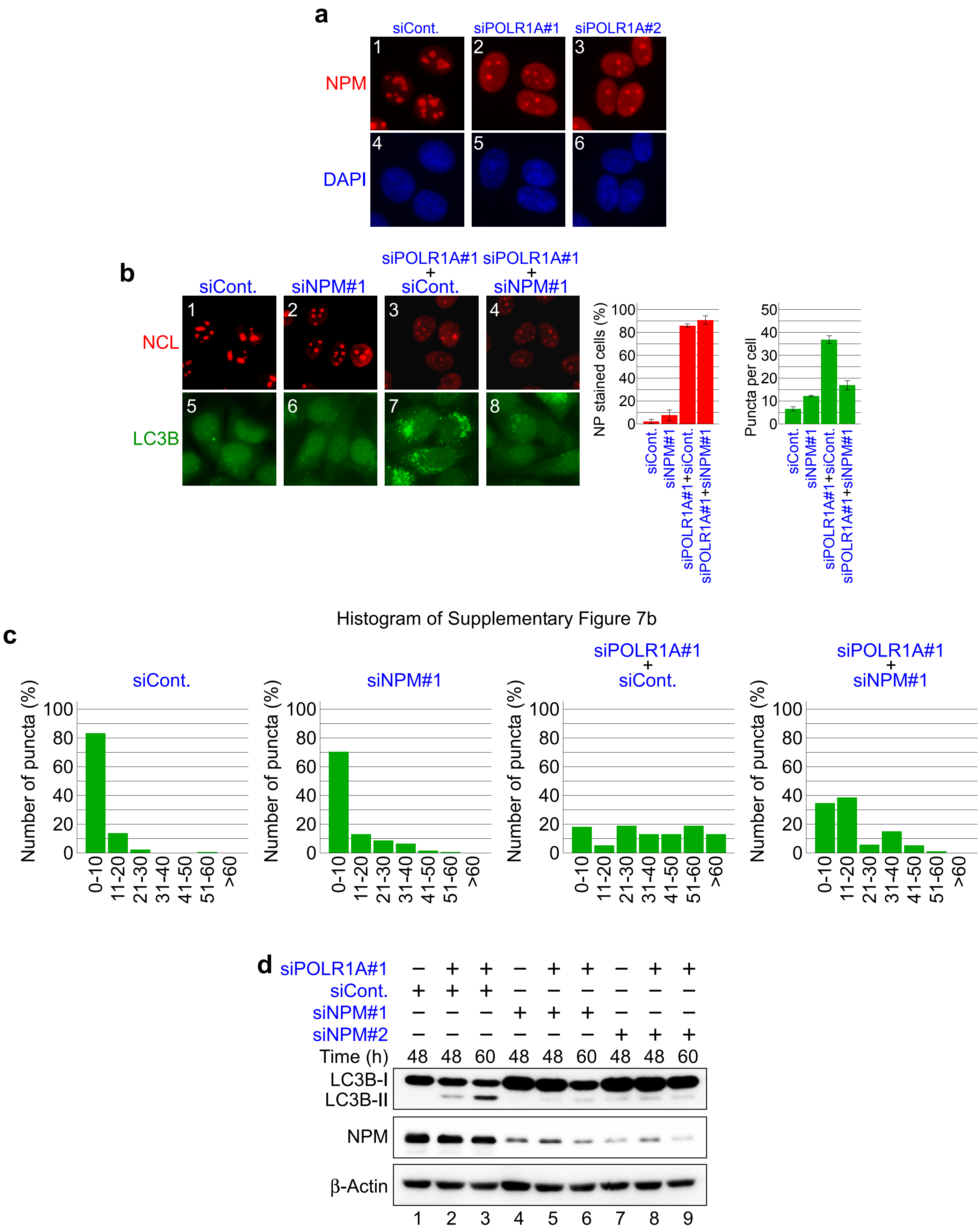
a
siCont.
siPOLR1A#1
siPOLR1A#2
NPM
DAPI
1
2
3
4
5
6
b
siCont.
siNPM#1
siPOLR1A#1 + siCont.
siPOLR1A#1 + siNPM#1
NCL
LC3B
1
2
3
4
5
6
7
8
NP stained cells (%)
0
20
40
60
80
100
Puncta per cell
0
10
20
30
40
50
siCont.
siNPM#1
siPOLR1A#1+siCont.
siPOLR1A#1+siNPM#1
Histogram of Supplementary Figure 7b
c
siCont.
siNPM#1
siPOLR1A#1 + siCont.
siPOLR1A#1 + siNPM#1
Number of puncta (%)
0
20
40
60
80
100
0-10
11-20
21-30
31-40
41-50
51-60
>60
d
siPOLR1A#1 – + + – + + – + +
siCont. + + + – – – – – –
siNPM#1 – – – + + + – – –
siNPM#2 – – – – – – + + +
Time (h) 48 48 60 48 48 60 48 48 60
LC3B-I
LC3B-II
NPM
β-Actin
1 2 3 4 5 6 7 8 9

Enlarged images of Figure 5b

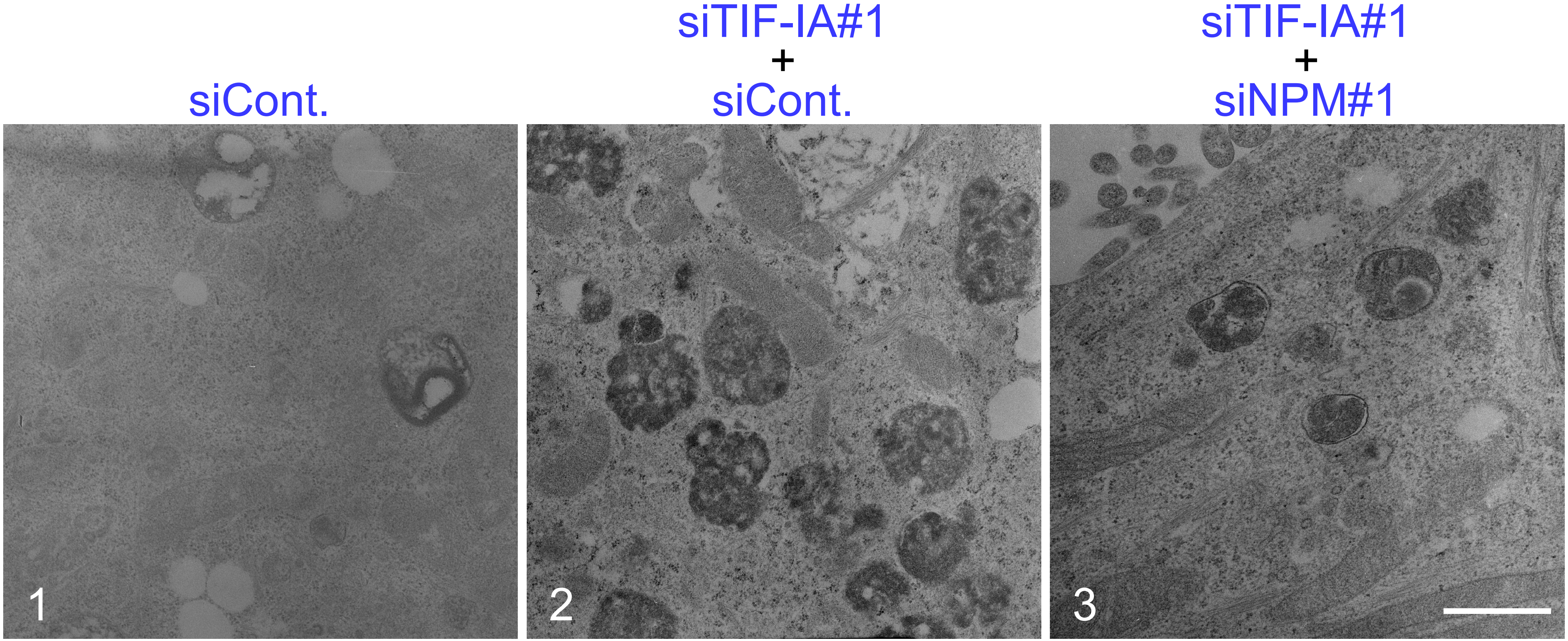

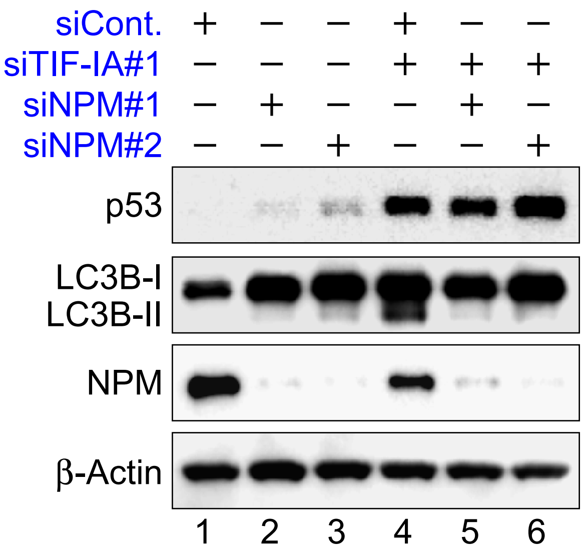
siCont. + − − + − −
siTIF-IA#1 − − − + + +
siNPM#1 − + − − + −
siNPM#2 − − + − − +
p53
LC3B-I
LC3B-II
NPM
β-Actin
1 2 3 4 5 6

a

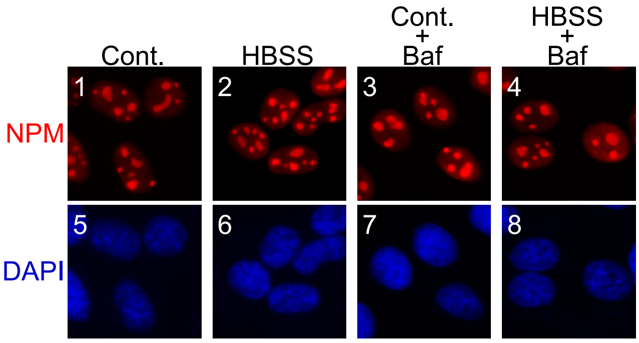

Histogram of Figure 6a

b

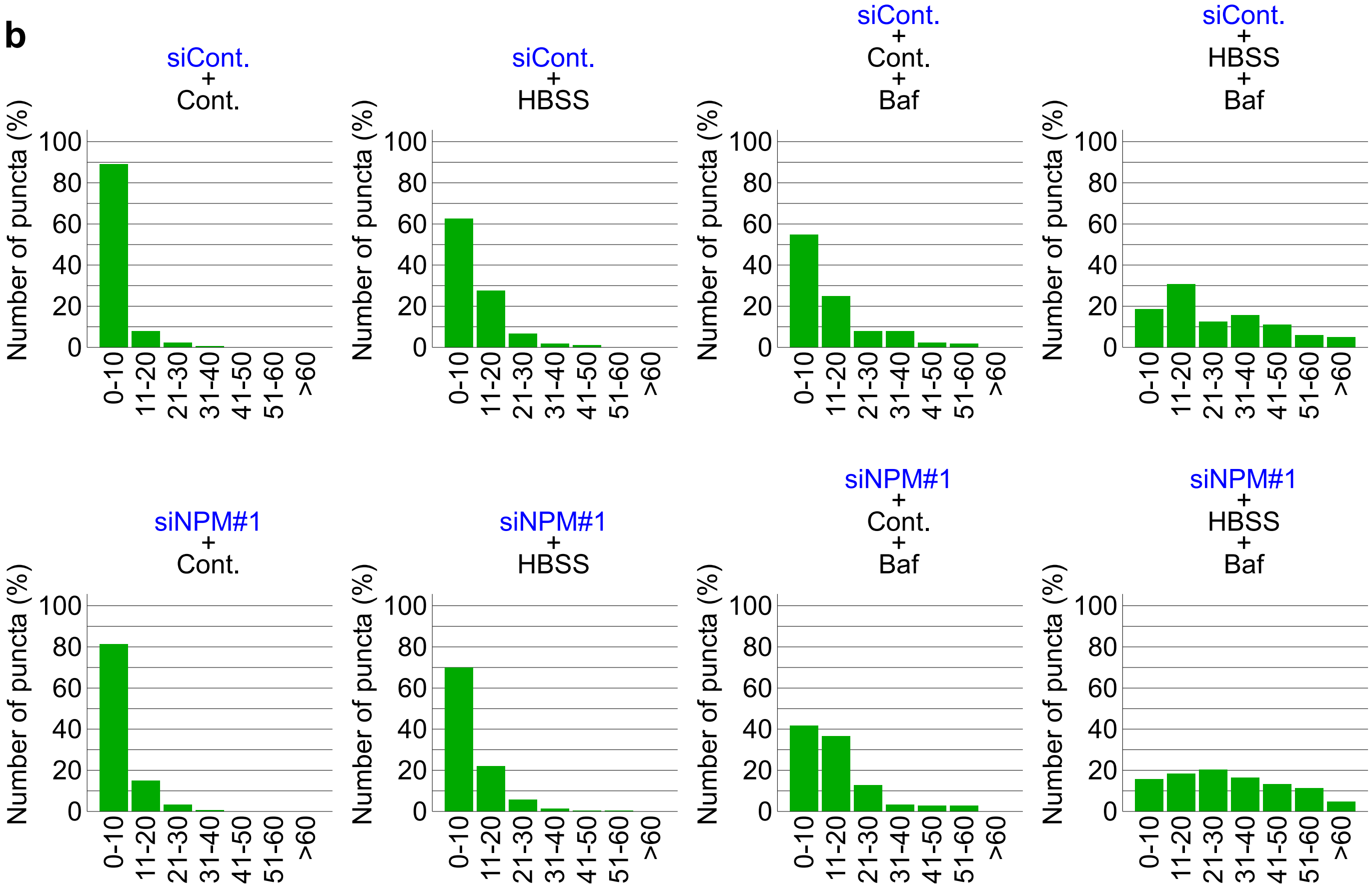

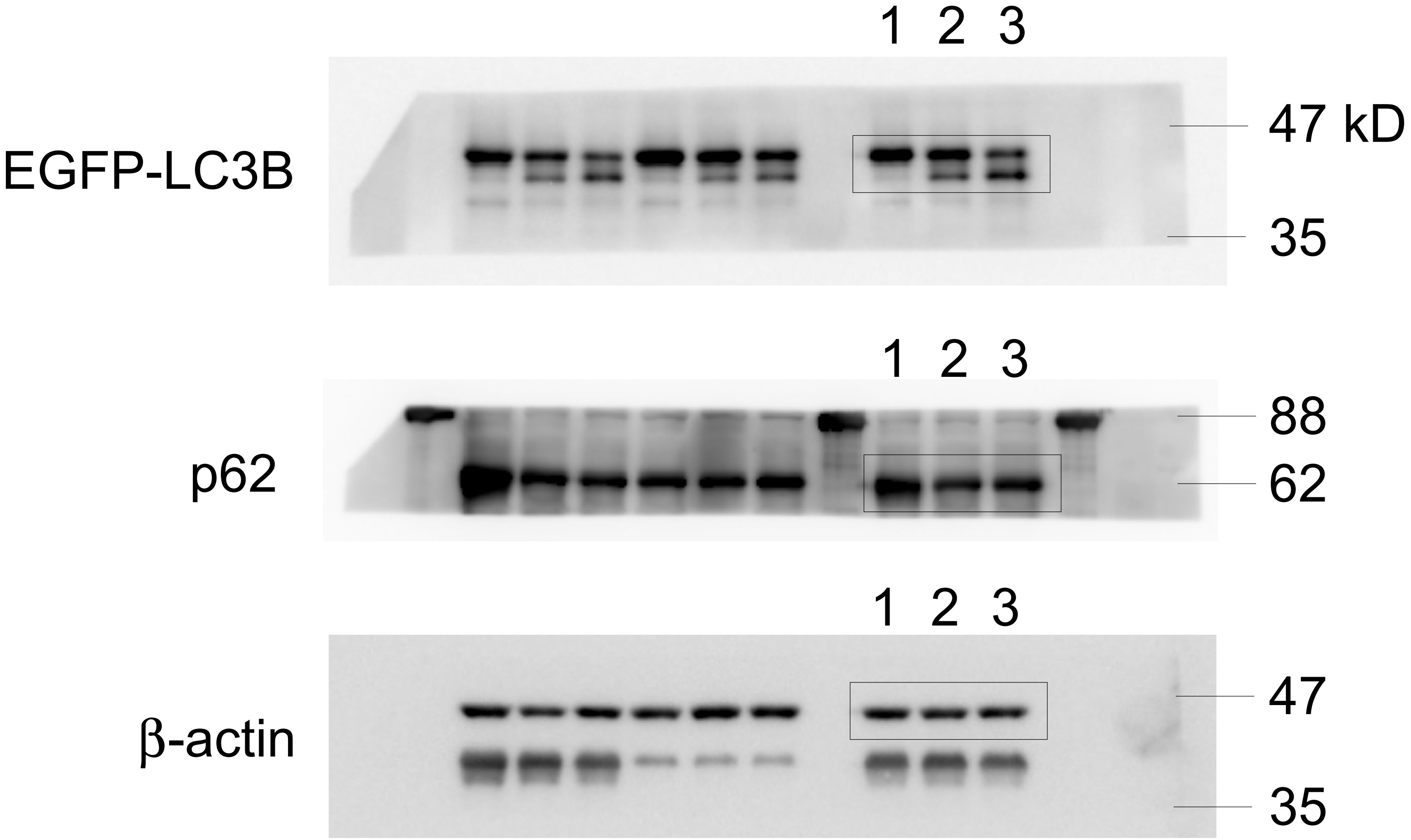

Fig.1b

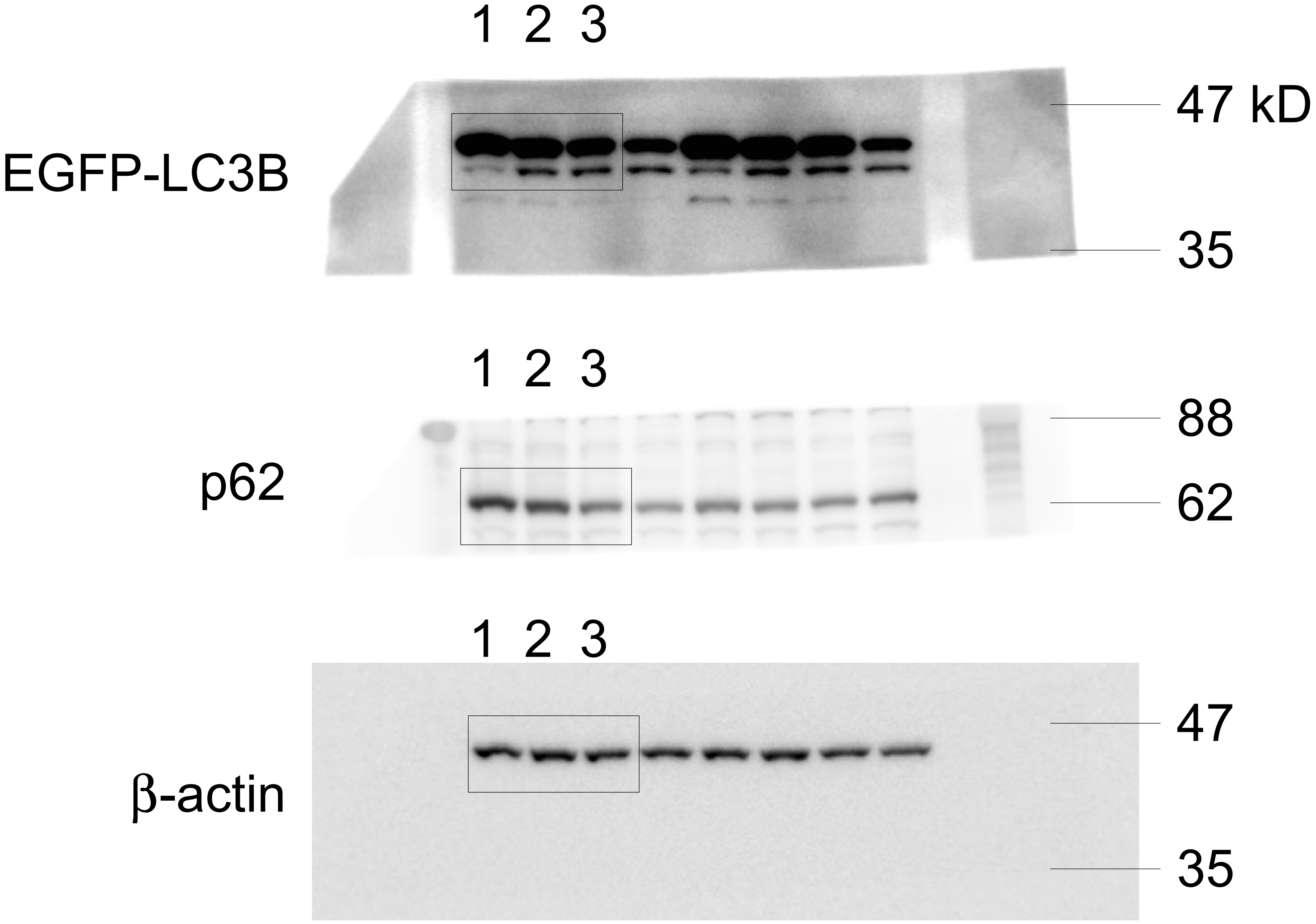

Fig. 1d

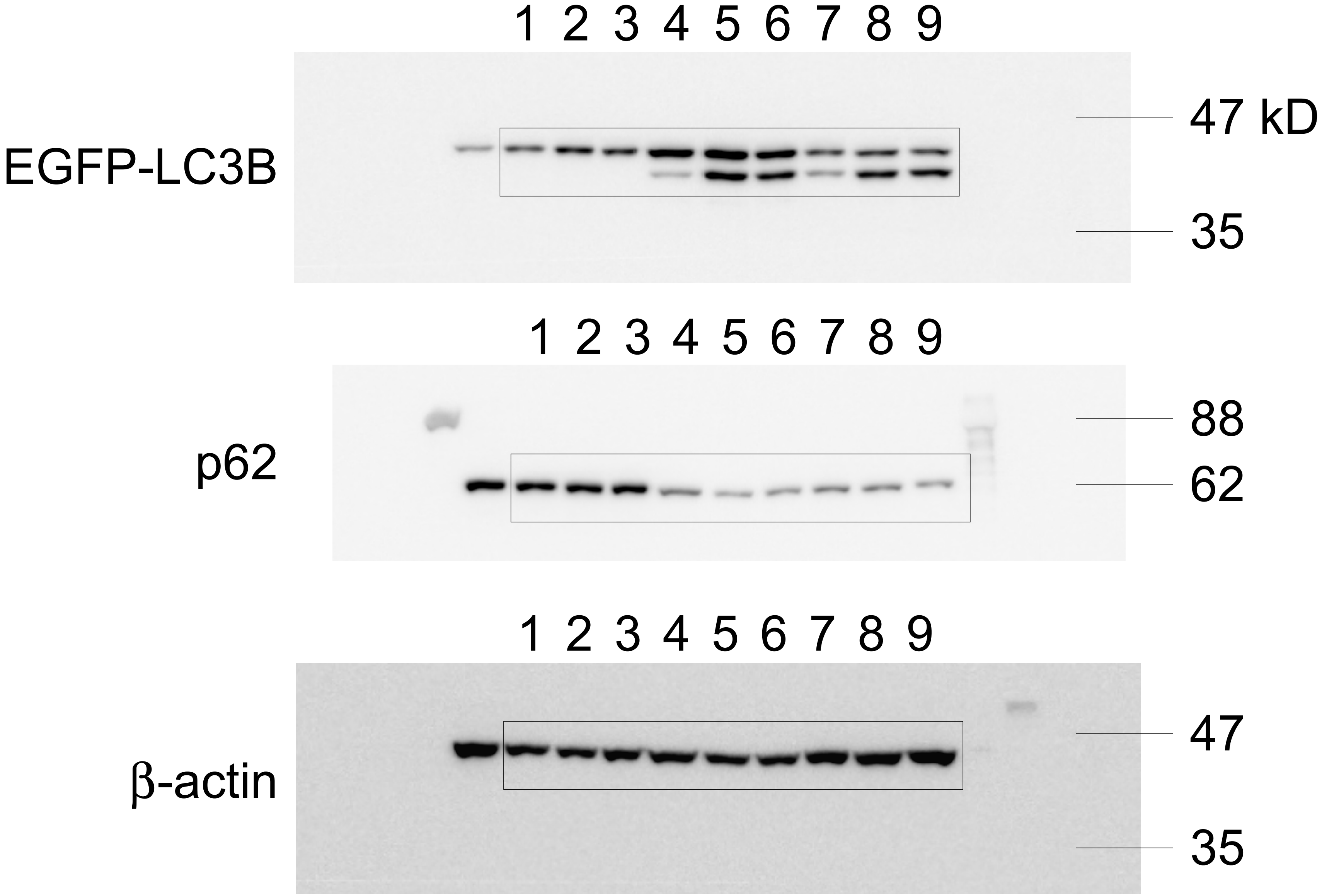
1 2 3 4 5 6 7 8 9
EGFP-LC3B
47 kD
35
1 2 3 4 5 6 7 8 9
p62
88
62
1 2 3 4 5 6 7 8 9
β-actin
47
35

Fig. 2b

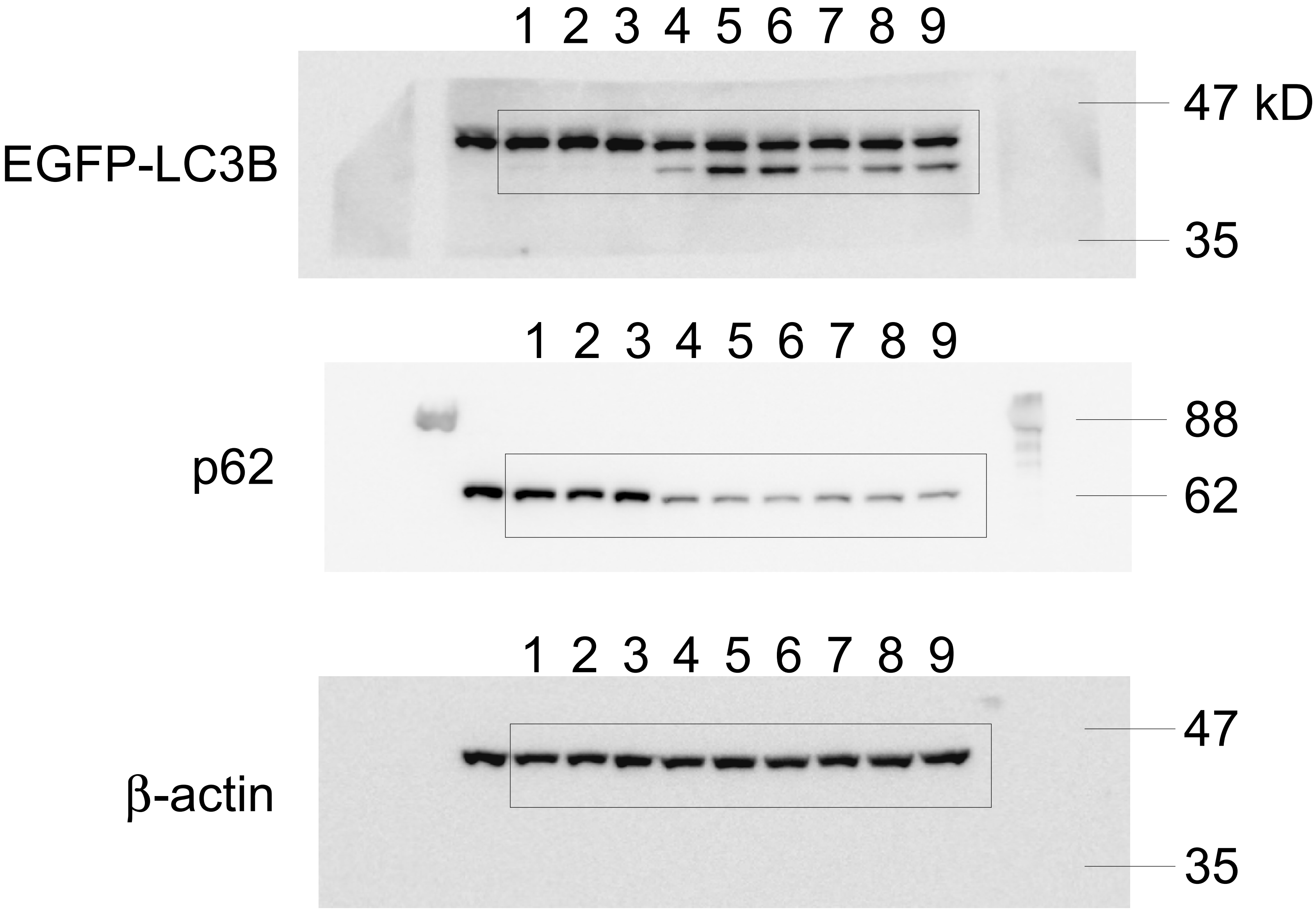

1 2 3 4 5 6 7 8 9
EGFP-LC3B
47 kD
35
1 2 3 4 5 6 7 8 9
p62
88
62
1 2 3 4 5 6 7 8 9
β-actin
47
35

Fig. 2d

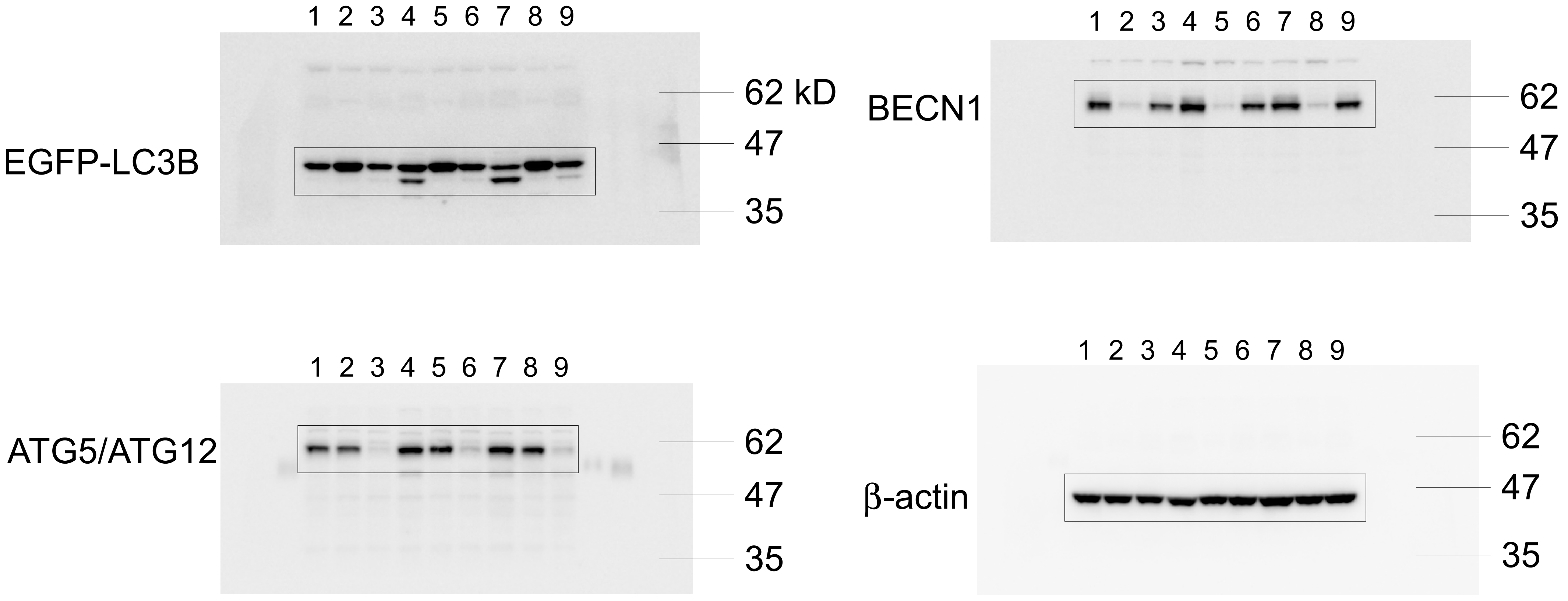

EGFP-LC3B
1 2 3 4 5 6 7 8 9
62 kD
47
35
BECN1
1 2 3 4 5 6 7 8 9
62
47
35
ATG5/ATG12
1 2 3 4 5 6 7 8 9
62
47
35
β-actin
1 2 3 4 5 6 7 8 9
62
47
35

Fig.3b

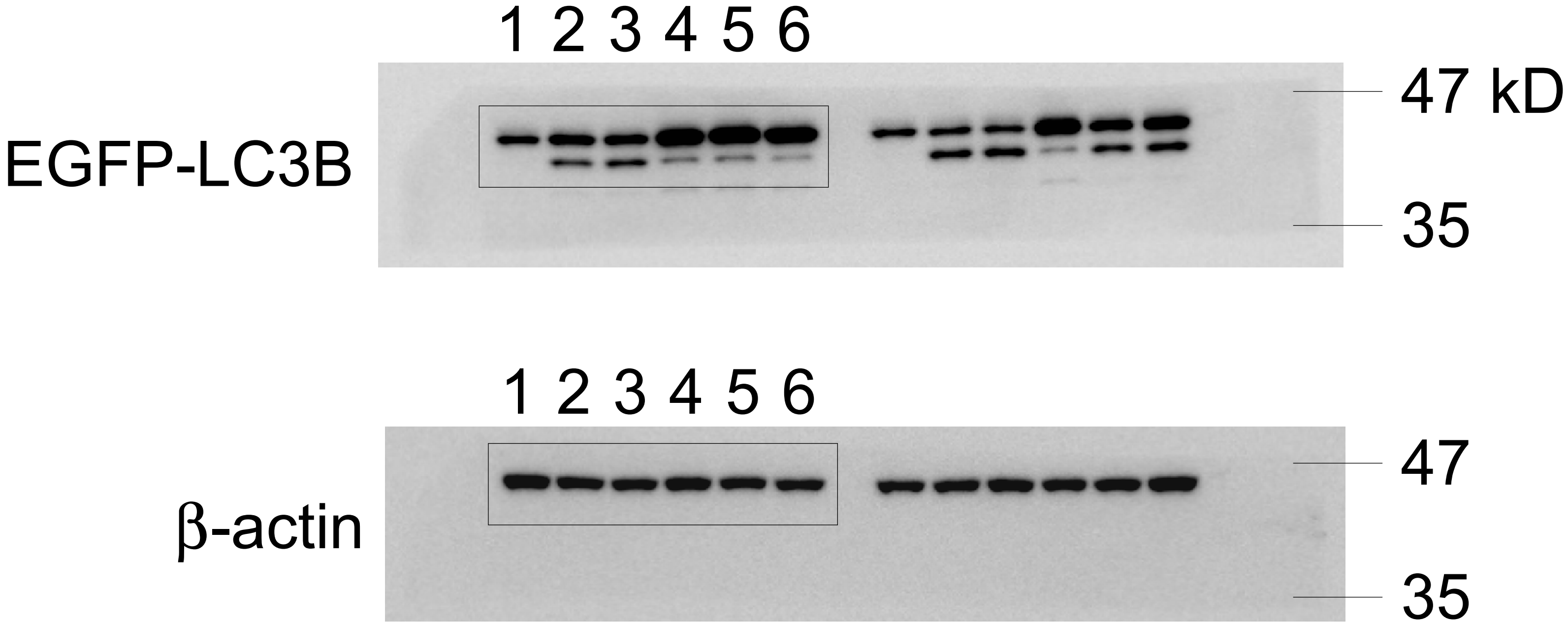

Fig. 3d

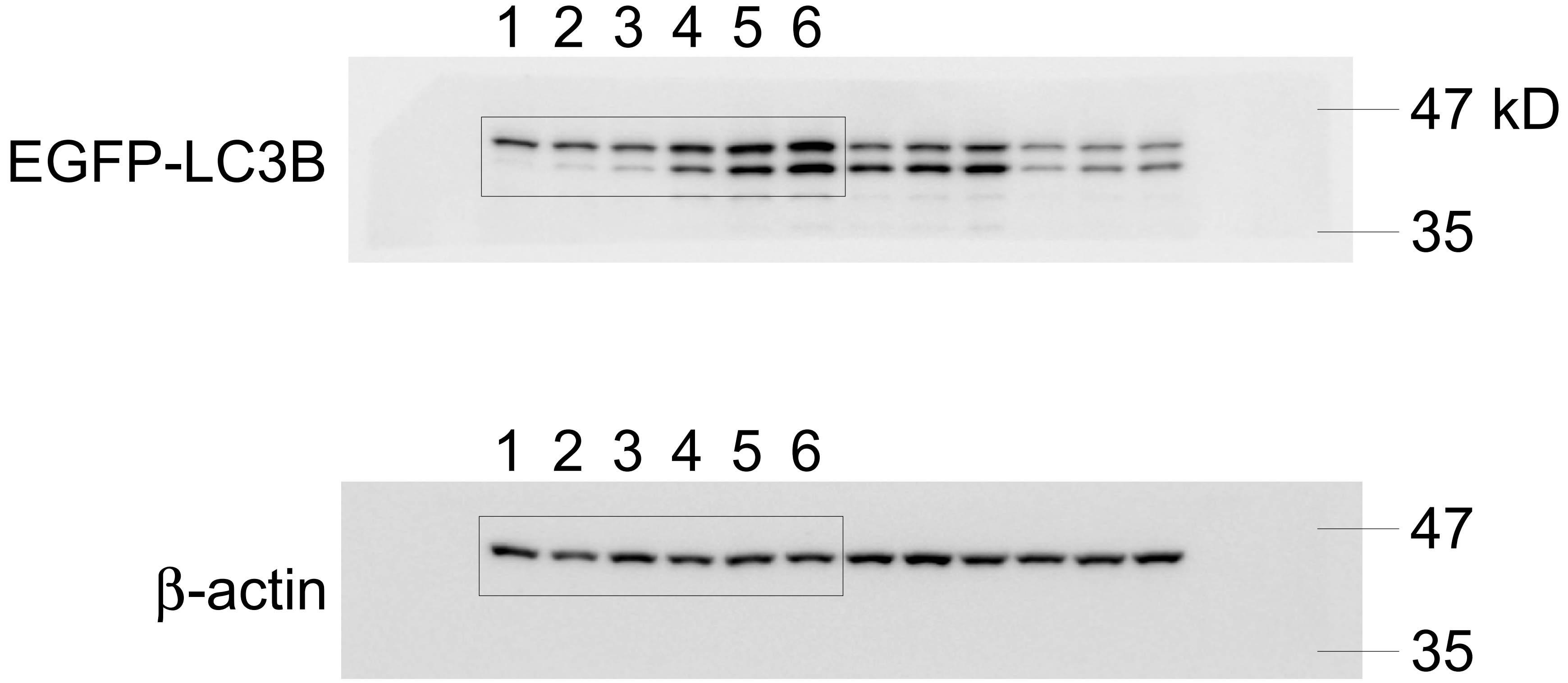

Fig. 3f

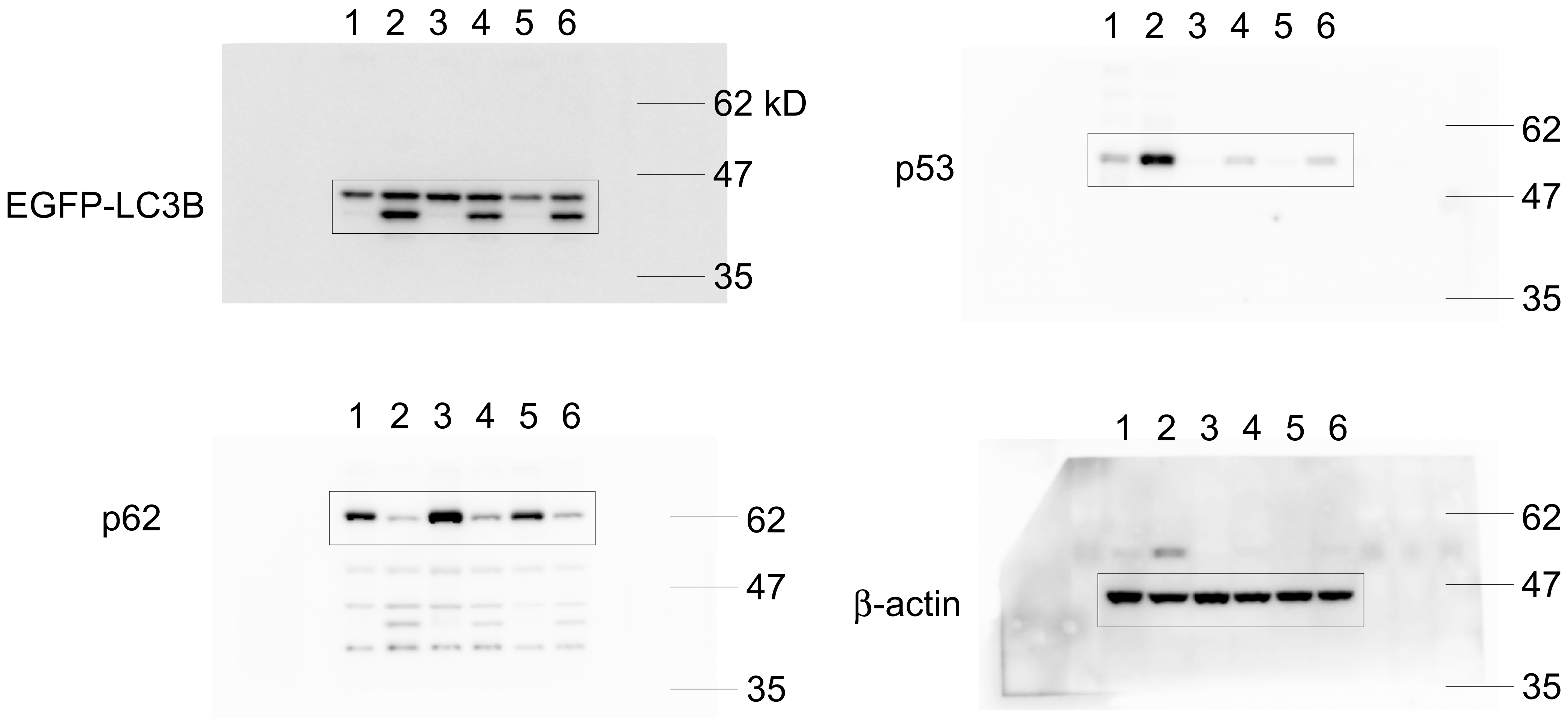
1 2 3 4 5 6
EGFP-LC3B
62 kD
47
35
1 2 3 4 5 6
p53
62
47
35
1 2 3 4 5 6
p62
62
47
35
1 2 3 4 5 6
β-actin
62
47
35

Fig. 4b

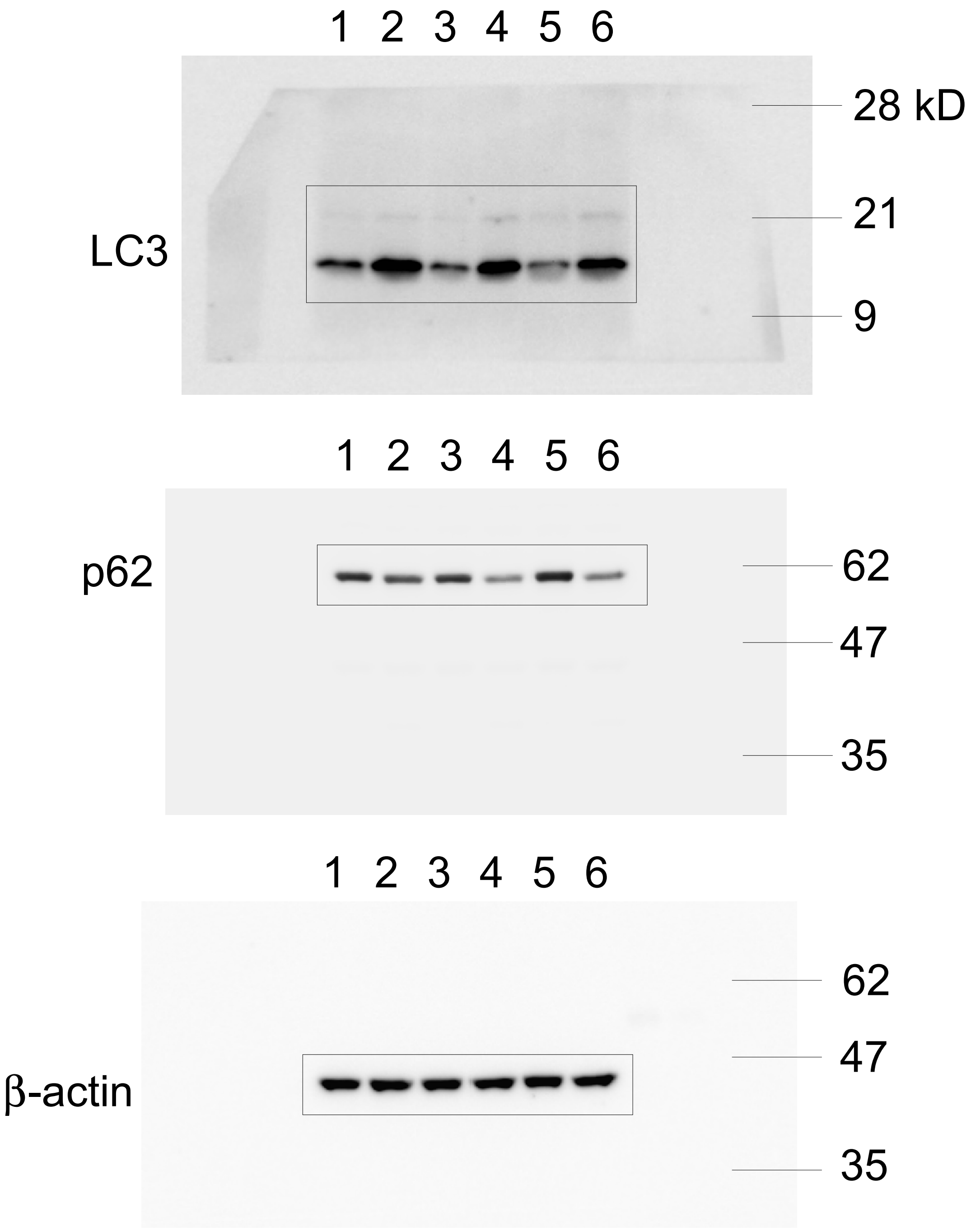

Fig. 4d

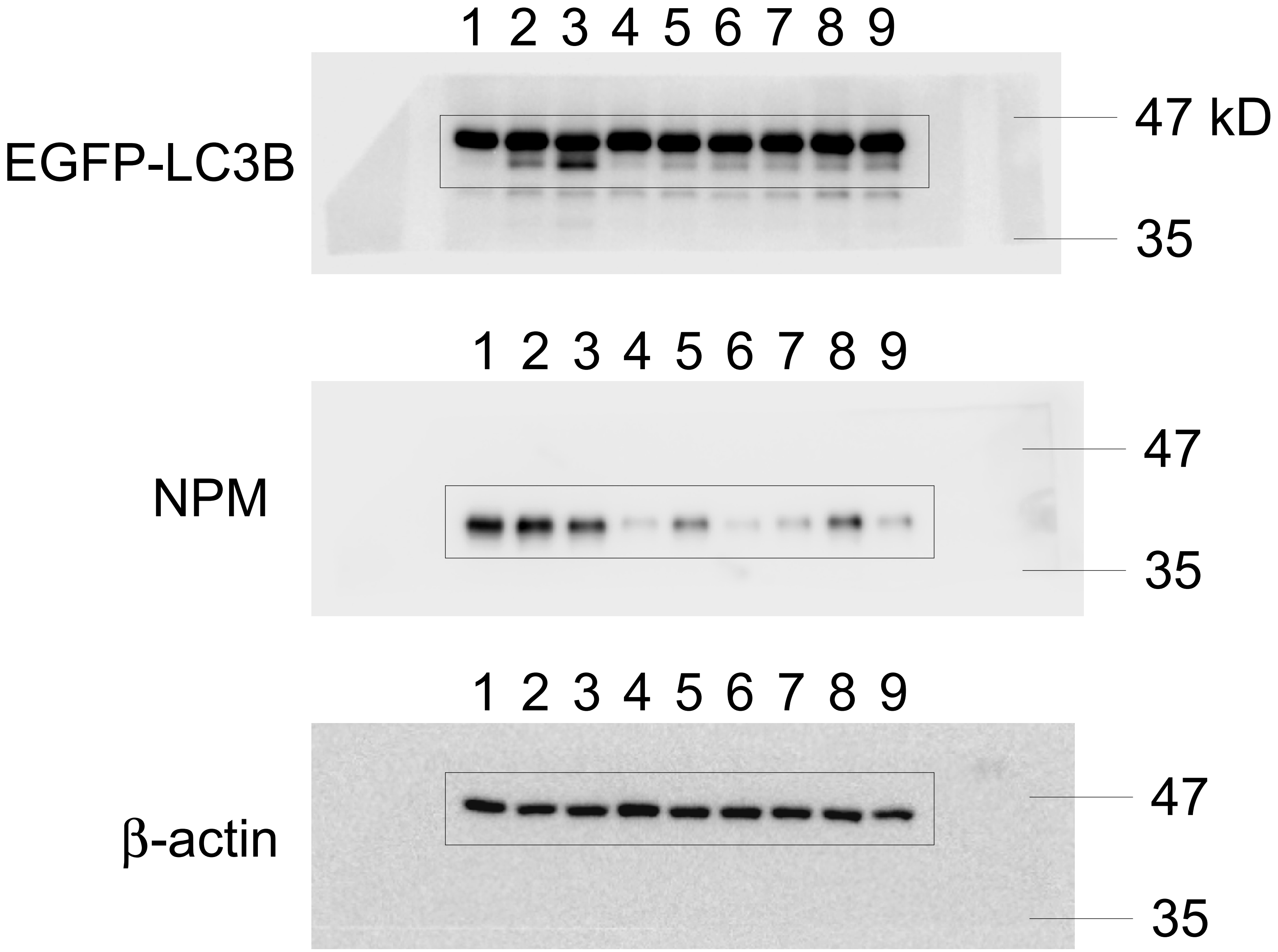
1 2 3 4 5 6 7 8 9
EGFP-LC3B
47 kD
35
1 2 3 4 5 6 7 8 9
NPM
47
35
1 2 3 4 5 6 7 8 9
β-actin
47
35

Fig. 5c

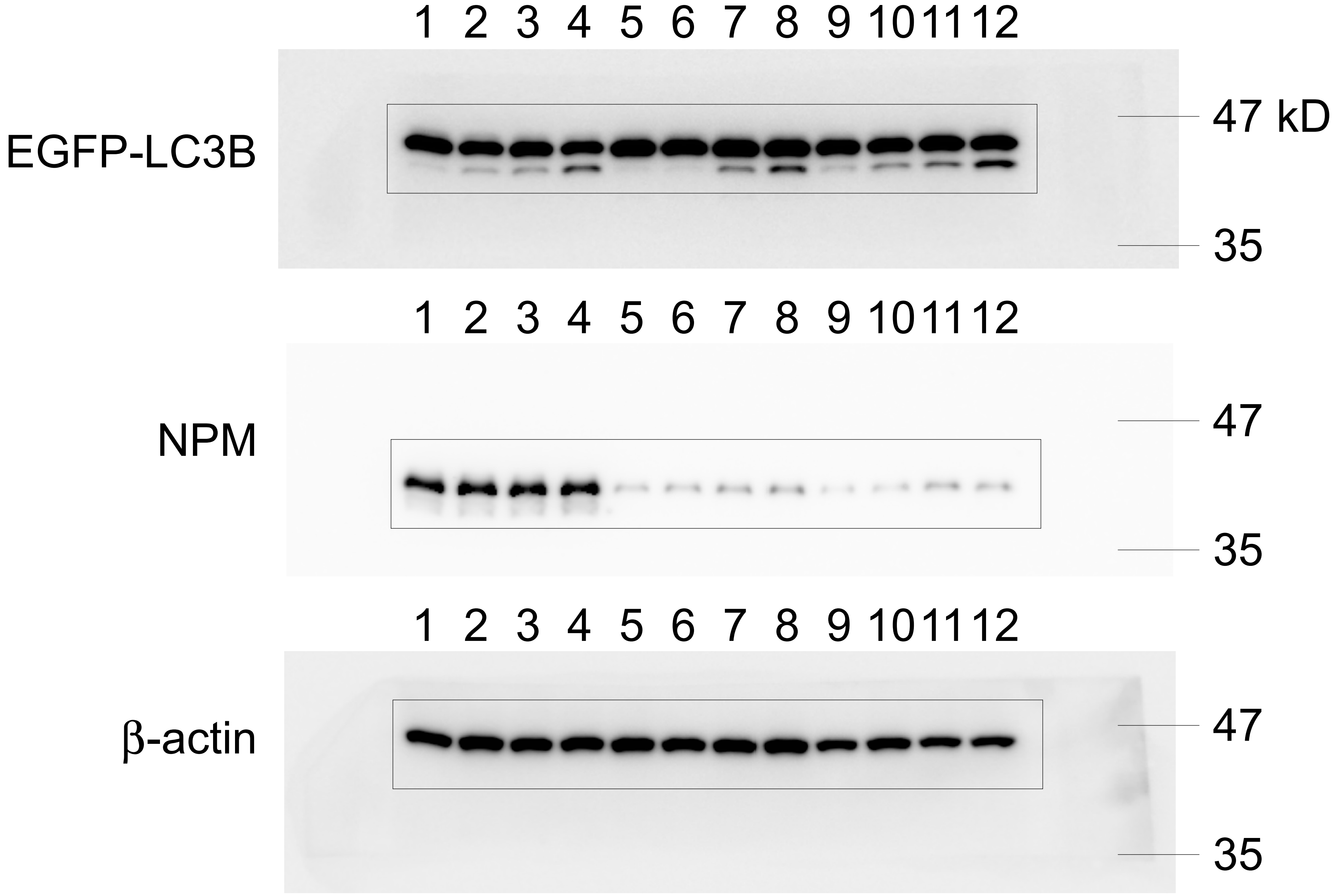

1 2 3 4 5 6 7 8 9 10 11 12
EGFP-LC3B
47 kD
35
1 2 3 4 5 6 7 8 9 10 11 12
NPM
47
35
1 2 3 4 5 6 7 8 9 10 11 12
β-actin
47
35

Fig. 6b
